# Neonatal Infections Caused by Multidrug-Resistant Bacteria: An Analysis of Prevalence, Risk Factors, and Therapeutic Implications—A Narrative Review

**DOI:** 10.3390/pathogens15050469

**Published:** 2026-04-26

**Authors:** Elena-Teona Coșovanu, Teodora Ana Balan, Eric-Oliviu Coșovanu, Silvia Ionescu, Costin Damian, Antoneta Dacia Petroaie, Elena-Adorata Coman, Mihaela Grigore, Demetra Socolov, Raluca Anca Balan, Luminita Smaranda Iancu, Irina Draga Căruntu, Ramona Gabriela Ursu

**Affiliations:** 1 “Grigore T. Popa” University of Medicine and Pharmacy, 16 Universitatii Street, 700115 Iasi, Romaniacostin-damian@umfiasi.ro (C.D.); irina.caruntu@umfiasi.ro (I.D.C.);; 2 “Cuza-Vodă” Clinical Hospital of Obstetrics and Gynecology, 700038 Iasi, Romania

**Keywords:** neonatal sepsis, multidrug resistance, antimicrobial resistance, NICU, Gram-negative bacteria, antimicrobial stewardship, infection prevention, neonatal therapeutics

## Abstract

Neonatal infections remain a leading cause of morbidity and mortality worldwide, particularly among preterm and low-birth-weight infants and in low- and middle-income countries. This burden has intensified with the global increase in multidrug-resistant (MDR) bacteria, especially in neonatal intensive care units, where prolonged hospitalization, invasive interventions, and exposure to broad-spectrum antibiotics promote colonization, transmission, and invasive infection. In this narrative review, we explore the epidemiology and microbiological characteristics of MDR bacterial infections in newborns, alongside their associated risk factors, diagnostic challenges, treatment outcomes, and prevention strategies. Across different settings, Gram-negative pathogens, particularly *Klebsiella pneumoniae*, *Escherichia coli*, and *Acinetobacter baumannii*, account for a substantial proportion of severe neonatal infections, whereas methicillin-resistant *Staphylococcus aureus* remains important in selected units. The risk of MDR infection is driven by a complex interplay of factors, ranging from maternal and perinatal exposures to the inherent immunological vulnerability of newborns, hospital-based transmission, antibiotic selection pressure, and structural deficiencies in healthcare infrastructure. Diagnosis remains challenging because clinical presentations are nonspecific and culture-based methods are constrained by low blood volumes, prior antimicrobial exposure, and delayed turnaround times. Treatment is increasingly complicated due to resistance to standard empirical regimens, substantial regional variation in susceptibility profiles, and limited neonatal pharmacokinetic and safety data for reserve agents. Current evidence mainly supports surveillance-informed empirical therapy, susceptibility-guided treatment adjustment, antimicrobial stewardship, and strict infection prevention measures. Future progress will require neonatal-specific clinical trials, harmonized surveillance systems, stronger molecular epidemiology, and more equitable access to microbiological diagnostics and effective treatment.

## 1. Introduction

Neonatal infections, particularly neonatal sepsis, remain among the leading causes of death and a major global health concern during the first 28 days of life. Despite substantial advances in perinatal and neonatal care, infectious diseases account for a substantial proportion of neonatal mortality worldwide, with the heaviest burden falling on low- and middle-income countries (LMICs) [1]. Globally, infections are estimated to contribute to approximately 23% of the 2.4 million neonatal deaths recorded annually [2]. The incidence of neonatal sepsis is estimated at 1–5 cases per 1000 live births, with the highest risk occurring in preterm infants and in those with very low birth weight [1].

The rapid global spread of antimicrobial resistance (AMR) further amplifies this burden, fundamentally altering both the epidemiology and the routine clinical management of infected newborns. In many neonatal sepsis cohorts, Gram-negative pathogens, particularly *Klebsiella pneumoniae* and *Escherichia coli*, predominate and are increasingly associated with multidrug-resistant phenotypes [1,3]. Across a wide range of healthcare settings, a substantial proportion of neonatal infections are now caused by multidrug-resistant bacteria, substantially narrowing therapeutic options and contributing to excess morbidity and mortality [1].

The clinical consequences of multidrug resistance are particularly evident in neonatal intensive care units (NICUs), where physiologically fragile infants are exposed to prolonged hospitalization, invasive procedures, and frequent antimicrobial use. Multidrug resistance, commonly defined as non-susceptibility to at least one agent in three or more antimicrobial classes, represents a major obstacle to effective treatment in neonatal practice. Bacterial pathogens of particular concern include *Klebsiella pneumoniae*, *Escherichia coli*, methicillin-resistant *Staphylococcus aureus* (MRSA), and *Acinetobacter baumannii*. Among these, extended-spectrum β-lactamase (ESBL)-producing *K. pneumoniae* has been reported at particularly high prevalence in some hospital environments, underscoring the intensity of resistance pressure in neonatal care settings [4].

Neonates are especially susceptible to invasive infection because of the combined effects of biological immaturity and healthcare-related exposure. Their underdeveloped immune system, including impaired neutrophil activity and reduced complement function, limits their ability to mount effective innate immune responses and increases vulnerability to colonization and subsequent infection by resistant pathogens [5]. This vulnerability is further compounded by immature skin and mucosal barriers, particularly in preterm infants, which facilitate the development of healthcare-associated infections [5,6]. In addition, early and often unavoidable exposure to broad-spectrum antibiotics, although frequently lifesaving, may disrupt the developing neonatal microbiota and promote colonization by resistant organisms [5,7].

AMR in neonatal care goes far beyond immediate clinical outcomes. It is increasingly recognized as a critical threat to global public health. Ultimately, it undermines healthcare resilience, worsens existing inequalities, and drives up economic costs, particularly in settings where resources are already severely constrained [1,8].

Although the threat posed by multidrug-resistant neonatal infections is increasingly acknowledged, the literature remains fragmented. Because much of the current evidence comes from single-center studies, outbreak reports, or specific pathogen investigations, LMICs often lack adequate representation. Furthermore, there is a noticeable gap in integrating microbiome research, genomic surveillance, and broader health-system perspectives, making a comprehensive and critically structured synthesis of the field particularly warranted.

Unlike previous reviews, this narrative review addresses this gap by providing a globally informed, clinically grounded, and context-sensitive overview of neonatal infections caused by multidrug-resistant bacteria. It examines the prevalence and geographic distribution of major resistant pathogens, evaluates maternal, neonatal, and healthcare-associated risk factors, and discusses current as well as emerging therapeutic and antimicrobial stewardship approaches. By integrating evidence across diverse healthcare contexts, this review aims to support clinical decision-making, strengthen stewardship efforts, and contribute to ongoing strategies to reduce the burden of antimicrobial resistance in neonatal care.

**Figure 1:**
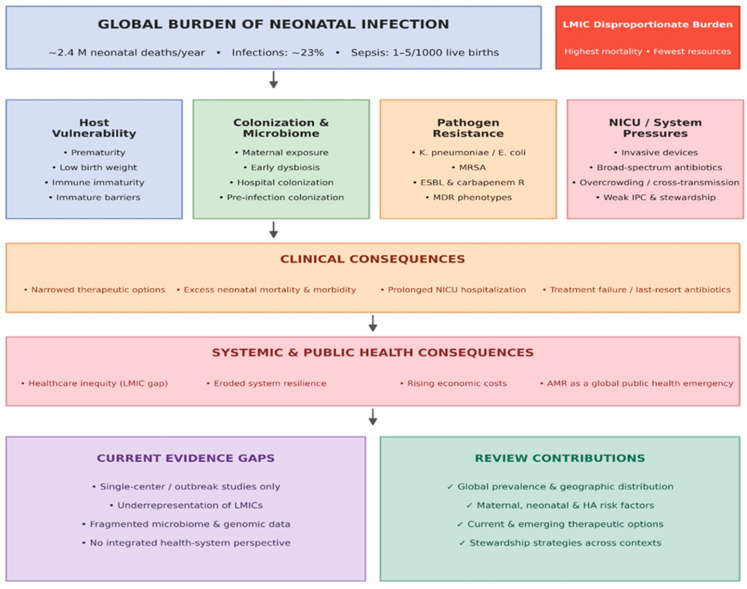
MDR Neonatal Infection: From Global Burden to Evidence Gaps and Review Contributions. Arrows indicate the conceptual progression from upstream determinants to clinical and public-health consequences, while colors distinguish the main thematic domains summarized in the figure.

Given the multifactorial nature of multidrug-resistant neonatal infection, the main determinants can be conceptualized as an interplay among host vulnerability, colonization dynamics, pathogen resistance, and NICU-related system pressures (Figure 1).

## 2. Methodological Approach

This review was designed as a narrative and integrative synthesis of contemporary literature on neonatal infections caused by multidrug-resistant (MDR) bacteria, aiming to integrate epidemiological, microbiological, and clinical evidence from diverse healthcare settings into a clinically relevant overview.

The primary literature search was conducted in PubMed/MEDLINE. The search strategy combined Medical Subject Headings (MeSH) and free-text terms related to neonates, bacterial infection, antimicrobial resistance, multidrug resistance, and neonatal intensive care, using Boolean operators to combine concepts. PubMed/MEDLINE was selected as the primary database because it provides broad coverage of peer-reviewed biomedical and clinical literature, indexes the neonatal, infectious diseases, and antimicrobial resistance fields in a standardized manner through MeSH terminology, and allows reproducible combinations of controlled vocabulary and free-text terms. For the clinical scope of this review, PubMed was considered the most appropriate source to capture the core literature on neonatal multidrug-resistant bacterial infections.

Although this article was designed as a narrative review, we adopted a structured search strategy resembling that of a systematic review in order to improve transparency, reproducibility, and selection consistency, while reducing the risk of arbitrary study inclusion. However, the objective remained interpretive and clinically integrative rather than exhaustive quantitative evidence synthesis.

Searches were limited to studies published between January 2010 and January 2026. The time window from 2010 to 2026 was chosen to emphasize contemporary evidence from the modern antimicrobial resistance era, including changes in neonatal intensive care practices, pathogen epidemiology, resistance mechanisms, and antimicrobial stewardship frameworks. Older studies were considered less comparable to current neonatal practice because of major temporal shifts in diagnostic methods, resistance patterns, and empirical treatment approaches.

**Figure 2:**
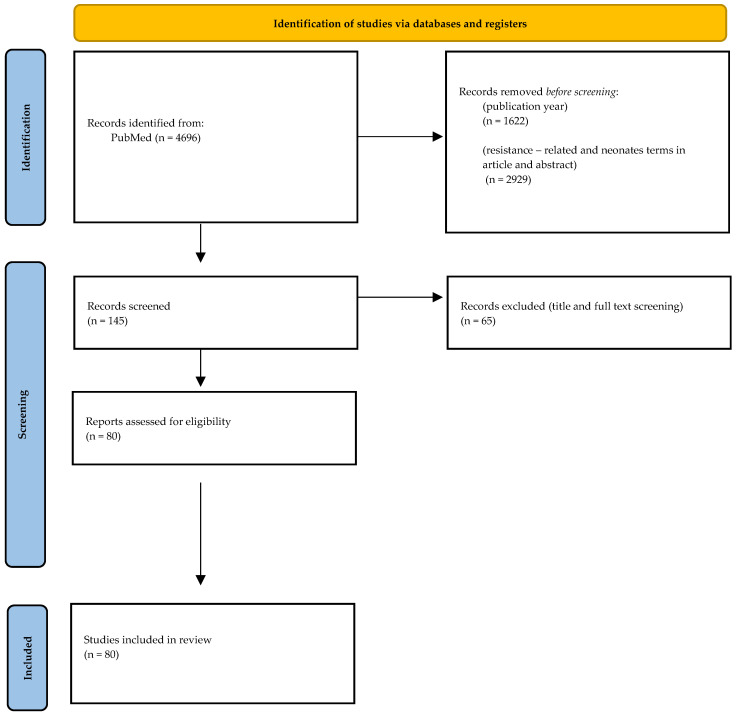
PRISMA 2020 flow diagram illustrating the PubMed/MEDLINE search and study selection process for the narrative review.

The initial broad PubMed search identified approximately 4696 records, which decreased to 3074 after restriction by publication year. A focused search strategy requiring resistance-related and neonatal terms in article titles and abstracts yielded 145 records, which underwent title and full-text screening, with 80 relevant articles selected for narrative synthesis. The complete PubMed/MEDLINE search string is provided in Supplementary Material S1. PRISMA flow diagram regarding the search and selection methods is shown as Figure 2.

Records were evaluated by the authors, and any uncertainties regarding inclusion were resolved through discussion and consensus.

Inclusion criteria: eligible publications included observational studies, clinical trials, systematic reviews, meta-analyses, and international guidelines addressing MDR bacterial infections in neonatal populations.

Exclusion criteria: studies were excluded if they focused on non-neonatal populations, non-bacterial pathogens, animal models, isolated case reports, or lacked specific relevance to antimicrobial resistance.

Given the considerable variation in study designs, patient populations, targeted pathogens, and reported outcomes, we chose to synthesize the findings narratively rather than conducting a quantitative analysis. The evidence was organized and interpreted thematically, rather than through a formal comparative assessment framework, in accordance with the objectives of the review, including epidemiology, pathogen distribution, risk factors, diagnostic challenges, therapeutic approaches, and prevention strate-gies.

## 3. Global Epidemiology and Prevalence of MDR Neonatal Infections

### 3.1. Burden of Neonatal Infections Worldwide

While neonatal sepsis is still a leading global cause of newborn illness and death, most available data derive from hospital and NICU-based studies in low- and middle-income countries rather than broader, population-level surveillance systems [9,10,11,12]. Comparisons across studies remain difficult because reported denominators vary widely, including NICU admissions, suspected sepsis episodes, culture-confirmed infections, and live births [9,10,12,13].

Despite these limitations, the burden remains substantial. In Delhi, DeNIS collaboration [9] reported sepsis in 14.3% of NICU admissions, with culture-positive sepsis accounting for 6.2% of admissions and 9.5 cases per 1000 live births. Aradhya et al. [14] found a sepsis incidence of 3.5% among admitted neonates in another Indian cohort, while Angulo-Zamudio et al. [12] reported neonatal sepsis in 4.0% of newborns in Mexico. Among suspected cases, culture confirmation rates were notably high, reaching 32.25% in Egypt according to Awad et al. [10], 19.2% in Tanzania according to Mkony [15], 40.4% in Ethiopia according to Geleta et al. [3] and 16% of blood cultures in Indonesia according to Putri et al. [11].

Across these studies, multidrug resistance represents not an occasional finding but a defining feature of neonatal sepsis epidemiology [3,9,10,16]. Overall MDR prevalence reached 77.0% in Egypt [10], 80.5% in Indonesia [16], 52.6% in Mexico [12] and 78.1% in Peru [17]. This burden is driven predominantly by Gram-negative organisms, particularly *Klebsiella* spp. and *Acinetobacter* spp., as reported by DeNIS collaboration [9], Gadisa et al. [18] and Ma et al. [19]. Even where pooled MDR rates were not calculated, extensive resistance to first-line agents such as ampicillin and gentamicin remained common [11,20,21].

The distinction between early-onset sepsis (EOS) and late-onset sepsis (LOS) remains important, although its epidemiological clarity varies across settings. In the Chinese systematic review, Yu et al. [20] found EOS to be more strongly associated with group B streptococcus and *Escherichia coli*, whereas LOS was more often linked to coagulase-negative staphylococci and *Klebsiella* spp. In contrast, Indian studies by DeNIS collaboration [9], Bandyopadhyay et al. [22] and Sharma et al. [23] showed substantial overlap between EOS and LOS pathogens, suggesting that healthcare-associated flora may influence infection patterns from very early life. Similar findings were reported from Egypt by Awad et al. [10], where Gram-negative organisms predominated in both EOS and LOS.

Mortality was high across studies, although estimates varied markedly. Reported mortality ranged from 9.5% in Uganda [24] and 10.1% in one Ethiopian cohort [4] to 37.5% in Egypt [10], 48.0% among positive culture cases in Delhi [9] and 59.7% in another Ethiopian cohort [3]. Although these estimates are not directly comparable, the overall pattern is consistent: settings with high MDR burden also tended to report worse outcomes. Findings from the DeNIS collaboration [9], Solomon [25] and Freitas [26] directly support this association. Beyond mortality, these resistant infections are also associated with prolonged hospital stays and more severe clinical complications [18,20,27].

### 3.2. Regional and Geographic Variability

**Figure 3:**
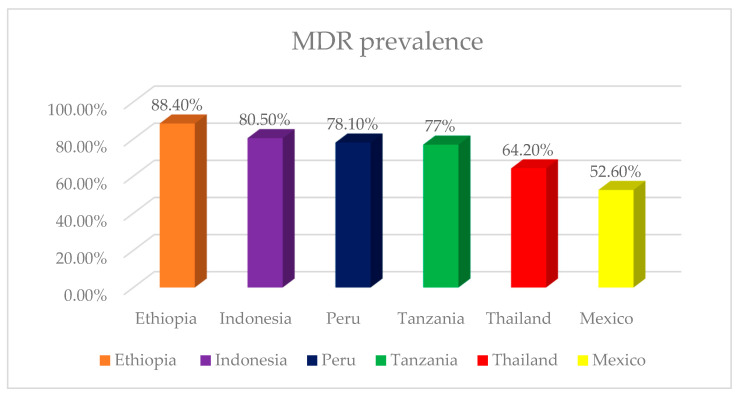
Global burden and dominant pathogen patterns in MDR neonatal infections. MDR prevalence by country/study, with mortality overlay.

The global burden of MDR neonatal infection is heterogeneous, with marked regional variation in prevalence, pathogen distribution, and onset-specific patterns (Figure 3). The available evidence suggests a broad contrast between low- and middle-income settings, where MDR neonatal sepsis is frequent and usually dominated by Gram-negative pathogens, and higher-income settings, where the reported MDR burden appears lower, especially in EOS [8,9,10,20,28] (Table 1). This contrast should nevertheless be interpreted with caution, as the literature is considerably denser for LMIC NICUs than for population-based studies in high-income countries, as noted by Yu et al. [20] and Darlow et al. [29].

In Africa, tertiary-care studies consistently report a high burden of resistant neonatal infection. Awad et al. [10] found that 33.3% of admitted neonates in Egypt had sepsis and that 77% of isolates were MDR. In Ethiopia, studies [3,18] reported very high MDR proportions, including 88.4% overall in one cohort and 95.7% among *Klebsiella pneumoniae* isolates in another. In Tanzania, Seni et al. [30] documented 77.8% MDR among bloodstream isolates, while in Uganda [24], high ampicillin resistance was reported despite a somewhat different pathogen distribution. Collectively, these studies indicate that MDR infection is a consistent feature of neonatal sepsis in African hospital settings.

South and Southeast Asia showed the most consistent evidence of a severe MDR burden. DeNIS collaboration [9] reported high mortality associated with resistant infections in Delhi, particularly those caused by *Acinetobacter* spp. and *Klebsiella* spp. Similar patterns were described in India [14,22,23]. In Pakistan, high MDR rates among Gram-negative neonatal isolates were reported [31,32]. In Thailand, Thatrimontrichai et al. [33] found that 64.2% of Gram-negative sepsis episodes were caused by MDR organisms, with a case-fatality rate of 37.6%. High MDR proportions were also reported from Cambodia and Indonesia [11,16,34]. Overall, MDR Gram-negative sepsis appears to be a defining feature of neonatal infection epidemiology across this region.

European data within the included literature were more limited and generally suggested a lower MDR burden than in many LMIC NICUs. Available studies [28,35,36] indicated that most EOS pathogens remained susceptible to empiric therapy, although resistance was greater in LOS. Spanish studies [28,37] highlighted the role of maternal transmission in neonatal *E. coli* infection, while ESBL-producing strains remained relatively uncommon. However, this pattern was not uniform, as Dedeić-Ljubović et al. [38] reported high rates of MDRO colonization and infection in a NICU in Bosnia and Herzegovina.

In Latin America, the burden appears substantial, although the evidence base remains smaller than that available for South Asia. In Mexico [13,39], hospital-associated infection was reported, with ESBL-producing Enterobacteriaceae being more frequent in LOS than EOS. More recent Mexican data [12] confirmed a high resistance burden, including MDR, extensively drug-resistant, and pandrug-resistant isolates. In Brazil, Freitas et al. [26] showed that MDR infection increased the odds of death in late-onset bloodstream infection and documented an outbreak of MDR *Acinetobacter baumannii*. In Peru [17], 78.1% of neonatal bloodstream isolates were MDR. These findings suggest that, in this region, NICU-associated infection is increasingly shaped by MDR pathogen epidemiology.

### 3.3. NICU-Associated MDR Infections

Available evidence strongly indicates that NICUs are major sites for the emergence and persistence of MDR neonatal infections [9,26,40,41]. Across studies, LOS consistently bears a heavier resistance burden than EOS and is dominated by typical healthcare-associated pathogens such as *Klebsiella pneumoniae*, *Acinetobacter baumannii* and other Enterobacterales [26,27,33,39].

Although only a minority of studies assessed colonization directly, those that did offered important insights. Lenglet et al. [40] linked rectal colonization to invasive ESBL-producing Gram-negative infection in Haiti. Dedeić-Ljubović et al. [38] reported high rates of both MDRO colonization and infection in Bosnia and Herzegovina, while Freitas et al. [26] documented colonization and invasive infection during a Brazilian NICU outbreak caused by MDR *Acinetobacter baumannii*.

Outbreak reports further demonstrate that NICU MDR epidemiology encompasses both epidemic and endemic transmission. Recurrent or suspected outbreaks were described for ESBL-producing *Klebsiella pneumoniae*, MDR *Acinetobacter baumannii*, and MRSA [26,40,42,43]. At the same time, several cohorts identified resistant pathogens as part of routine NICU microbiology rather than as isolated outbreak events [9,27,41]. This suggests that many units experience continuous MDR circulation, with outbreaks superimposed on a background of endemic transmission.

Environmental contamination also appears to play a relevant role. Odoyo et al. [44] detected MDR ESKAPEE organisms on 12.6% of hospital surfaces in newborn and maternity areas, including incubators, cots, sinks, and equipment. Other studies linked transmission to overcrowding, invasive devices, prolonged hospitalization, and deficiencies in infection prevention [10,27,45]. Maternal and perinatal reservoirs may also play a contributing role, particularly in EOS [24,37].

Taken together, the evidence shows that MDR neonatal infection is concentrated in hospital and NICU settings. It is especially prominent in LMICs and is driven largely by resistant Gram-negative pathogens, particularly in LOS [9,10,11,33]. Regional differences are evident, but the evidence base remains uneven, with the strongest data coming from South Asia, Africa, and selected Latin American cohorts, whereas data from high-income countries and population-based studies remain limited [17,20,29].

## 4. Socioeconomic and Health System Determinants

Across the reviewed studies, it is evident that multidrug-resistant (MDR) neonatal infections stem from more than just direct clinical exposures. They are firmly rooted in broader health-system conditions that dictate prevention, diagnosis, treatment, and referral pathways [27,46]. As a result, prior antimicrobial exposure, invasive procedures, and delayed therapies must be understood in the context of structural determinants, such as limited facility capacity, inconsistent access to laboratory services, poor stewardship and ongoing social disadvantages [33,47,48]. This pattern is particularly evident in low- and middle-income settings, where resistant neonatal infection is repeatedly linked to constrained health infrastructure [11,40,49].

Health-system capacity is a central determinant, as it influences both transmission risk and the quality-of-care following infection. Across hospital-based studies, overcrowding, understaffing, high patient acuity and infection prevention gaps recur as conditions that facilitate the persistence and spread of resistant pathogens, particularly in NICU and referral settings [9,46,47]. Several studies also suggest that pressure on referral hospitals, fragmented surveillance, and variable institutional resources weaken timely detection and response, suggesting that resistance is partly a systems-level problem rather than solely a prescribing issue [11,17,48].

Restricted access to microbiological diagnostics is one of the clearest pathways through which structural weakness affects management. In more constrained settings, delayed turnaround times, low culture yield, weak laboratory support and interruptions in supplies limit timely confirmation and susceptibility-guided treatment [15,21,27,32]. As a result, clinicians often continued empiric therapy despite incomplete microbiological evidence, with implications for both overtreatment and delayed optimization of care [24,49].

Antibiotic availability and regulation represent a related yet distinct systemic determinant. The reviewed studies describe recurrent stewardship problems, including overuse of broad-spectrum agents, dependence on empiric regimens and weak regulatory control in some settings [24,48,50,51]. Several reports also indicate that access to effective treatment may be limited by availability, affordability, or policy constraints, particularly for reserve agents [10,29,31,42,52]. Treatment failure or delayed effective therapy should therefore not be understood solely as clinical problems at the bedside, but also as consequences of wider regulatory and pharmaceutical inequality.

Evidence regarding rural-urban disparities is less consistent and warrants cautious interpretation. Some studies suggest that geographic inequity operates through under-resourced district catchment areas, rural residence, and referral dependence rather than through a simple urban-rural gradient [18,46]. For instance, Gadisa et al. [18] reported that most positive cases occurred among rural residents, whereas Seni et al. [30] did not identify rural versus urban residence as a significant predictor of bloodstream infection or mortality. The more consistent signal is therefore uneven access to diagnostic and treatment capacity across levels of care [21].

Home births and perinatal care gaps may further increase vulnerability to MDR infection before hospital admission. The clearest direct association comes from Ethiopia, where Gadisa et al. [18] linked home birth to MDR *Klebsiella pneumoniae* sepsis and also identified adverse perinatal factors such as multiple vaginal examinations, difficult labor, and prolonged rupture of membranes. Other studies support the relevance of early care gaps by documenting home births attended by traditional birth attendants, unhygienic cord care, prelacteal feeding and absent maternal screening or obstetric services in some settings [13,39,53,54]. These findings suggest that vulnerability to resistant infection may be compounded prior to NICU admission by deficiencies in safe delivery practices, maternal screening, and referral continuity.

More broadly, the reviewed literature indicates that the MDR burden is concentrated in populations already affected by health inequities. Poverty, limited access to care, weak surveillance, and low-resource neonatal services recur as contextual features in studies from high-burden settings [34,40,45,48,49]. Several datasets also include outborn or referred infants, suggesting that fragmented care pathways and delayed access to high-capacity facilities may worsen both exposure risk and early management [21,47,53]. Taken together, the evidence suggests that MDR neonatal infection is not merely a microbiological problem, but also a manifestation of inequitable health-system performance. The strongest support concerns low facility capacity, uneven microbiological access, weak stewardship and antibiotic regulation, and broader social inequities, particularly in LMIC settings, whereas evidence for independent rural-urban effects remains less robust [27,30,40,47,48,49,50].

## 5. Microbiological Landscape of MDR Pathogens

### 5.1. Predominant MDR Bacterial Species

In the reviewed literature, Gram-negative bacteria predominate in MDR neonatal infections. In contrast, resistant Gram-positive pathogens are reported far less frequently and generally account for a smaller proportion of the overall burden [49,55]. Among Gram-negative organisms, *Klebsiella pneumoniae* appears most consistently as the leading pathogen, particularly in hospital-associated and late-onset neonatal sepsis, where it is frequently associated with multidrug resistance and ESBL production [19,25,47,54].

*Escherichia coli* is also a major pathogen, but its role is more variable. It remains important in neonatal sepsis, especially in broader or early-onset cohorts, yet it is less consistently identified as the dominant MDR organism in NICU-based settings compared with *K. pneumoniae* [47,49,56]. *Acinetobacter baumannii* is less uniformly prevalent, but it is clinically significant due to its extensive resistance profile and propensity for clonal spread in hospital settings [9,26]. *Pseudomonas aeruginosa* is identified less frequently but is typically associated with challenging treatment and poor clinical outcomes [11,33,51].

Among Gram-positive organisms, the main MDR pathogen is *Staphylococcus aureus*, particularly MRSA, whereas vancomycin-resistant enterococci are reported less consistently and rarely emerge as dominant pathogens [9,22,24,47]. Less frequent pathogens, such as *Serratia* spp., *Burkholderia cepacia*, and *Salmonella enterica*, appear mainly in localized outbreaks or specific cohorts, rather than as consistently dominant pathogens [57,58,59,60].

### 5.2. Mechanisms of Antimicrobial Resistance

The most consistently reported resistance mechanism across the reviewed studies is ESBL production, particularly among Enterobacterales and especially in *K. pneumoniae* and *E. coli* [19,47,61]. This is clinically significant as it compromises the activity of third-generation cephalosporins, which remain part of empiric treatment in many neonatal settings [4,34,62].

Carbapenem resistance represents an additional major layer of concern, although it is less uniformly characterized at the molecular level. Where genetic data were available, studies identified enzymes such as OXA-23 in *A. baumannii*, VIM-1 in *Serratia* isolates, and NDM-1 in carbapenem-resistant *K. pneumoniae* [26,57,63]. Many studies, however, reported carbapenem non-susceptibility without detailed molecular confirmation, which limits the precision of mechanistic characterization [46,64].

By contrast, the roles of efflux pumps, porin loss, and biofilm formation could not be confidently synthesized, as these mechanisms were rarely investigated directly [49,65,66,67]. In Gram-positive organisms, the main mechanism presented is methicillin resistance in *S. aureus*, while vancomycin resistance in enterococci is reported less consistently [9,24].

### 5.3. Genomic Epidemiology and Molecular Surveillance

Molecular surveillance is represented in the reviewed literature but remains unevenly applied. Whole-genome sequencing was used in only a minority of studies, but when applied it allowed more detailed analysis of resistance genes, genomic relatedness, and resistome structure [49,57,59]. Even without WGS, several studies documented clonal spread using lower-resolution methods, particularly during NICU outbreaks involving *K. pneumoniae* and *A. baumannii* [26,60,68].

The reviewed studies also suggest a role for horizontal gene transfer, although direct evidence remains limited. The strongest evidence derives from reports of plasmid-mediated ESBL genes, experimentally transferable resistance determinants, and mobile genetic elements such as class 1 integrons and IncL/M plasmids [28,57,68]. However, plasmid and integron characterization was not routine, and resistome-level analysis remained uncommon, particularly for Gram-positive organisms [22,49,59,69].

Molecular data further suggest that transmission within neonatal units is shaped by both clonal spread and mobile genetic elements, although high-resolution genomic surveillance remains inconsistently implemented [49,57,68].

## 6. The Neonatal Microbiome and MDR Infection Risk

The reviewed literature addresses the relationship between the neonatal microbiome and MDR infection primarily through colonization and transmission patterns rather than direct microbiome profiling [11,38,40,70,71,72]. Within this context, intestinal colonization is more consistently reported than specific microbiome profiles [11,38,40,72].

Neonatal exposure to MDR organisms has been documented through both maternal and hospital-based sources. Maternal-neonatal transmission, including ESBL-producing organisms, has been reported in early-onset settings [37,73]. By contrast, late-onset MDR infection is more frequently associated with hospital acquisition, cross-transmission, contaminated hands or equipment, and prolonged hospitalization [9,11,38,60,63,74].

Among clinical factors discussed in this context, the reviewed studies do not demonstrate a consistent association between mode of delivery and MDR outcomes [9,18,20,22,23,41,63,67]. Breastfeeding has been discussed in relation to beneficial gut colonization, and lower MDR Gram-negative colonization has been reported among breastfed infants [22,35,70,71].

Broad-spectrum antibiotic exposure has also been documented in neonatal settings characterized by MDR colonization or infection [53,72,74,75]. In addition, immature host defenses, incomplete microbiome establishment, and deficient mucosal barriers are discussed in relation to increased susceptibility to MDR infection [38,60,70,71].

Overall, the most consistent finding may be the relevance of colonization dynamics during hospitalization. Several reports describe progressive intestinal colonization during hospitalization as being associated with subsequent infection [11,38,40,72].

## 7. Risk Factors for MDR Neonatal Infections

### 7.1. Maternal and Perinatal Risk Factors

Maternal infection or colonization represents a plausible upstream source of resistant early-onset infection, particularly in studies involving ESBL-producing Gram-negative organisms [23,37,71]. However, most studies captured these exposures through clinical proxies such as fever, urinary tract infection, chorioamnionitis, or suspected genital tract colonization rather than systematic microbiological screening, which limits direct inference [12,24,63].

Antenatal antibiotic exposure has been examined less frequently; nevertheless, the available studies suggest that it may contribute to resistant early-onset disease. The clearest signal emerges from Sharma et al. [23], in which maternal antibiotic exposure was independently associated with ESBL-producing early-onset sepsis. Premature rupture of membranes (PROM) is more consistently supported and appears to be the strongest maternal-perinatal risk factor across the reviewed studies, particularly in relation to resistant early-onset Gram-negative infection [1,10,16,23,39].

### 7.2. Neonatal Risk Factors

Among neonatal factors, the most consistent risks are prematurity and low birth weight. This finding recurs across hospital-based cohorts, outbreak reports, and reviews, especially in studies of MDR Gram-negative sepsis [18,19,22,27,42]. Prematurity appears to be the most reproducible host-related determinant, likely reflecting both biological fragility and greater exposure to intensive care [28,49,71].

Low birth weight and very low birth weight follow a similar pattern, although their effects are often difficult to disentangle from those of prematurity. Several studies have linked lower birth weight to more severe infection or poorer outcomes [20,26,27,76]. Immune immaturity, by contrast, is typically presented as a biological explanation rather than a directly measured predictor [19,49,71,76].

**Figure 4:**
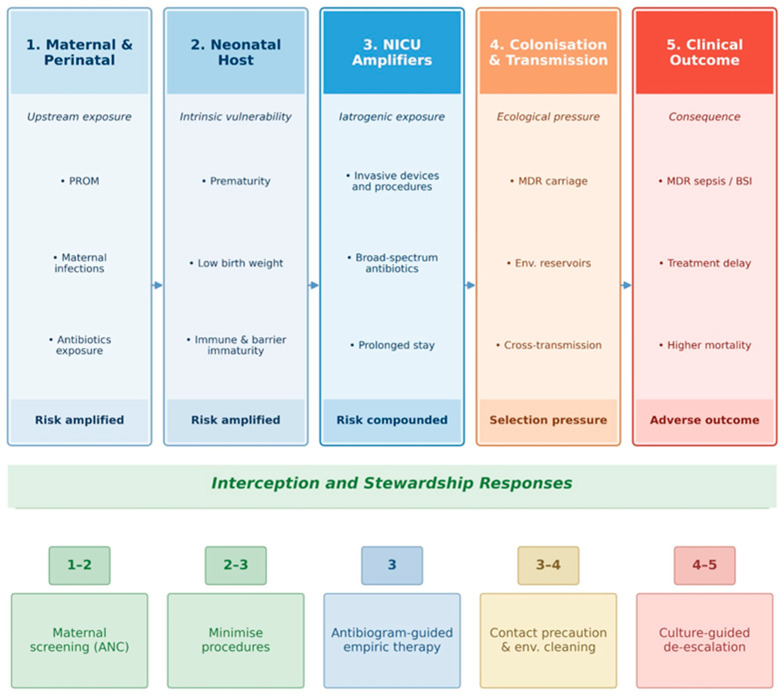
Clinical Pathway from Risk Exposure to MDR Neonatal Sepsis. Arrows indicate the sequential progression from upstream exposure and host vulnerability to colonization, transmission, and clinical outcome, while colors distinguish the main pathway domains and corresponding stewardship responses.

The progression from perinatal risk exposure to colonization, invasive infection, delayed effective therapy, and infection-control response can be conceptualized as a clinical pathway relevant to NICU practice (Figure 4).

### 7.3. Healthcare-Associated Risk Factors

Healthcare-associated factors are the strongest and most consistently reported risk factors. Across NICU-based studies, invasive interventions consistently increase exposure to MDR organisms in already vulnerable neonates, a pattern that is particularly evident in late-onset sepsis [20,22,27,47,49].

Mechanical ventilation is a frequently reported marker of high-risk intensive care, although its effect is not consistently quantified across studies [20,22,27,52,67]. Central venous and umbilical vascular catheters are more consistently documented and appear to represent the most reproducible healthcare-associated risk determinants [20,27,35,47,71].

Parenteral nutrition is reported less often, but when assessed, it appears to identify neonates at particularly elevated risk [20,23,33,71]. Prolonged hospitalization is among the most evident contextual risk factors and likely reflects cumulative exposure to invasive procedures, colonized environments and repeated antibiotic pressure [11,19,27,47,49,53,58].

### 7.4. Antibiotic Exposure and Stewardship Failures

Antibiotic pressure is among the most consistently identified themes in the reviewed literature and operates at both the individual and system levels [35,49,50,76,77]. Empirical broad-spectrum therapy is particularly common in LMIC NICUs and referral hospitals, where high background resistance rates frequently drive escalation beyond narrow first-line regimens [11,14,23,27,34].

Prolonged or inappropriate therapy is also recurrently described as a driver of MDR selection; however, many studies address it conceptually rather than measuring it in a standardized manner. Nevertheless, several reports directly associate prior antibiotic exposure, multiple antibiotic courses, irrational use, or inappropriate empirical treatment with resistant infection and poor outcomes [21,27,35,50,53].

The most consistently identified risk factors across the reviewed literature are PROM, prematurity, low birth weight, vascular catheter use, prolonged hospitalization, and broad-spectrum antibiotic exposure [18,20,27,76].

## 8. Clinical Manifestations and Diagnostic Challenges

### 8.1. Clinical Spectrum of MDR Neonatal Infections

Across the reviewed studies, multidrug-resistant (MDR) neonatal infection is reported primarily as sepsis or bloodstream infection, whereas meningitis, pneumonia, and device-associated infection are described less consistently and are frequently embedded within broader sepsis cohorts [9,11,14,22,41]. The dominant clinical presentation is therefore neonatal sepsis, with other manifestations generally representing organ-specific extensions of invasive infection rather than clearly distinct syndromes.

Sepsis is the most consistently documented presentation and is typically reported in clinically unstable neonates presenting with shock, thrombocytopenia, coagulopathy, renal dysfunction, or feeding intolerance [19,23,27,41]. Several studies also distinguish clinically suspected from culture-confirmed sepsis, indicating that bedside diagnosis remains central, as the clinical burden consistently exceeds the microbiologically confirmed burden [9,31,32,46].

Meningitis and pneumonia are also part of the reported spectrum. Some cohorts documented clinically meaningful proportions of meningitis or respiratory involvement [14,22,41]. Other studies identified device exposure as a risk context rather than as a distinct syndrome [18,27,65].

### 8.2. Diagnostic Limitations

One of the most recurrent themes across these studies is the gap between clinical suspicion of infection and microbiological confirmation, a delay that directly affects treatment decisions and the interpretation of outcomes [27,32,40,46]. The first major limitation is the nonspecific nature of neonatal presentation. Symptoms such as temperature instability, poor feeding, lethargy, respiratory distress, abdominal distension, jaundice, irritability, and seizures are common but do not reliably distinguish MDR infection from susceptible infection or from noninfectious clinical deterioration [23,27,39,40,63].

A second limitation is the low sensitivity and delayed yield of blood culture. Several studies reported low culture positivity rates, culture-negative sepsis, delayed reporting, and reduced organism recovery following prior antibiotic exposure [9,32,40,46,49]. In one study, recognition of MDR infection was delayed by 48–72 h, illustrating how culture-dependent diagnostic workflows can delay effective treatment in critically ill neonates [27]. Limited blood volume further reduces diagnostic sensitivity and complicates repeated sampling [23,26,40,49].

### 8.3. Emerging and Advanced Diagnostic Tools

The reviewed studies support only a limited and cautious role for advanced diagnostics. Molecular methods were predominantly used to characterize resistance mechanisms, confirm unusual organisms, or support outbreak investigations, rather than to replace culture-based methods in routine clinical care [19,26,59,63,68]. Whole-genome or hybrid sequencing similarly appears as a high-resolution investigative tool rather than a standard frontline diagnostic method [25,49,59].

Evidence supporting multiplex PCR panels in routine neonatal care is limited within this dataset; therefore, stronger conclusions are not warranted. Biomarkers such as CRP, procalcitonin, leukocyte indices, platelet count, and lactate may support suspicion of infection, but they do not reliably differentiate MDR from non-MDR infection and cannot substitute for microbiological confirmation [19,23,27,63].

### 8.4. Barriers to Implementation

The main barriers to advanced diagnostics are structural. Cost, limited laboratory infrastructure, and insufficient trained personnel are recurrently reported across the reviewed studies, especially in high-burden, resource-constrained settings [17,24,26,46,49]. Several studies relied on external laboratories or specialized expertise for confirmatory testing, suggesting that successful implementation depends not only on equipment availability but also on technical and interpretive capacity [26,59,78]. Ethical considerations were less thoroughly addressed, although the burden of additional blood sampling represents a clinically relevant concern in neonates [49].

Taken together, the reviewed evidence indicates that MDR neonatal infection is clinically dominated by sepsis, while meningitis and pneumonia remain important but less consistently defined manifestations [9,14,22,41]. The main diagnostic challenge lies in the combination of nonspecific presentation, low-yield culture-based confirmation, and restricted blood volume, all of which delay etiologic diagnosis and complicate treatment, while advanced diagnostics currently contribute most value in resistance characterization and selected investigative contexts rather than in routine frontline care [27,32,40,49] (Table 2).

## 9. Therapeutic Implications and Treatment Challenges

### 9.1. Empirical Therapy in the MDR Era

Across the reviewed studies, empirical treatment remains largely based on conventional neonatal regimens—most often ampicillin or benzylpenicillin combined with gentamicin—for early-onset sepsis, while broader combinations are more frequently employed in late-onset sepsis or nosocomial infection [13,33,35,39,41]. Several studies suggest that these regimens may provide inadequate coverage in settings with a high prevalence of MDR Gram-negative pathogens, particularly *Klebsiella pneumoniae* and *Acinetobacter* spp., based on microbiological resistance data [4,10,11,14,21,30].

However, the reviewed evidence does not support indiscriminate expansion to universal broad-spectrum empirical therapy. Drawing on epidemiological reasoning and stewardship principles—rather than controlled trial data—several authors recommend that empirical treatment be guided by local resistance patterns, surveillance data, and risk-stratified algorithms [11,27,35,40,64]. This position reflects expert consensus in the absence of prospective comparative evidence. The central challenge is therefore not whether to initiate antibiotics promptly, but rather how to calibrate empirical choices while minimizing unnecessary exposure to reserve agents.

### 9.2. Targeted Antimicrobial Therapy

Once microbiological results become available, the reviewed studies support de-escalation or redirection of therapy based on culture and susceptibility findings, rather than continuation of fixed empirical protocols [18,27,33,41]. Antibiogram-guided prescribing emerged as a recurring theme in treatment optimization throughout the reviewed literature [11,14,19,21,32].

Combination therapy was frequently reported, but predominantly as a pragmatic response to severe or highly resistant infection, rather than as a strategy validated through comparative trials. It should be noted that most reviewed studies lacked control arms or prospective designs, and observed outcomes with combination regimens cannot therefore be reliably attributed to the combination itself. A microbiologically rationalized example was the use of ceftazidime-avibactam plus aztreonam for MDR *Stenotrophomonas maltophilia*, where the combination was justified by the organism’s beta-lactamase profile [58]; however, this represents case-level evidence that cannot be broadly generalized. Based on available data, combination therapy should be regarded as a situational rather than a routine strategy, supported primarily by microbiological rationale and clinical reasoning [18,58,72].

### 9.3. Pharmacokinetic and Safety Challenges

Uncertainty in pharmacokinetics and pharmacodynamics (PK/PD) remains a significant challenge in the treatment of MDR infections in neonates, particularly for repurposed or last-line agents, as acknowledged across multiple reviewed studies [49,64,79]. Given that neonatal drug metabolism changes rapidly with maturation, the evidence base supporting the dosing of many reserve antibiotics remains limited, particularly for preterm or critically ill neonates [29,49,80]. This reflects a recognized limitation of the current literature rather than a gap addressed within it.

Regarding flomoxef, Darlow et al. [29] showed through PK modelling that drug disposition is influenced by body weight and postnatal age, supporting age-stratified dosing. For colistin, Nakwan et al. [52] and Ambreen et al. [31] similarly documented substantial PK/PD uncertainty in critically ill neonates through observational data. Dosing recommendations derived from these studies should be interpreted with caution, as microbiological susceptibility alone does not guarantee adequate drug exposure in this population, and extrapolation to broader neonatal groups should be approached carefully.

Nephrotoxicity was the most consistently reported adverse effect, particularly with colistin, with rates of 5.8% [52] and 5.2% [31], based on observational data. Other toxicities—including electrolyte disturbances and possible class-related adverse effects of salvage agents—were reported less consistently across studies [49,79,80], and variability in monitoring protocols limits meaningful direct comparisons.

### 9.4. Novel and Adjunctive Therapies

Within the reviewed literature, the available evidence for therapeutic innovation pertains to newer or repurposed antibiotics rather than experimental biological platforms [19,29,49,79]. Flomoxef, fosfomycin, tigecycline, and ceftazidime-avibactam were the agents most frequently discussed, primarily due to retained in vitro or observational activity against resistant Gram-negative organisms. Nevertheless, neonatal efficacy and safety data for these agents remain scarce, and their current clinical use relies largely on extrapolation from adult pharmacokinetic studies or small case series [58,64,80].

Bacteriophage therapy, antimicrobial peptides, CRISPR-based antimicrobials, and passive immunotherapy were either minimally represented or entirely absent as established neonatal treatment modalities in the evidence reviewed [41,50,66,81]. Their potential future relevance has been discussed in the emerging literature; however, no direct neonatal evidence was identified within the scope of this review. Adjunctive strategies such as rectal surveillance and probiotics were discussed primarily as stewardship or preventive tools rather than treatments for established invasive infection [27,40,70]; their use in this context is supported by indirect evidence and expert consensus rather than controlled neonatal trial data.

Taken together, the reviewed studies indicate a mismatch between conventional empirical regimens and current resistance patterns in high-burden settings, particularly where MDR Gram-negative organisms predominate [11,14,21]. Susceptibility-guided therapy refinement and local antibiogram-informed prescribing appear to be the most consistently emphasized recommendations across the reviewed literature [14,19,32]. Broader stewardship principles—including the avoidance of indiscriminate use of reserve agents—are supported by epidemiological reasoning and expert consensus, but remain to be evaluated in prospective neonatal trials.

## 10. Infection Prevention and Control Strategies

### 10.1. Antimicrobial Stewardship in Neonatal Units

Across the reviewed studies, antimicrobial stewardship in neonatal care is presented primarily as a surveillance-informed strategy aimed at optimizing empirical therapy, reducing unnecessary broad-spectrum exposure, and aligning prescribing with local resistance patterns [11,14,21,35,55,56,64]. The most consistent recommendation is that neonatal antibiotic policies should be adapted to unit-specific epidemiology, particularly in NICUs with a high burden of MDR Gram-negative infection [4,9,10,32].

Some studies describe targeted strategies such as cephalosporin restriction, rectal screening to guide de-escalation during outbreaks, and carbapenem-sparing approaches in ESBL-endemic settings [23,29,40,79]. However, the literature more frequently emphasizes stewardship principles than formally structured neonatal stewardship programmes with standardized outcome reporting [35,47,71].

### 10.2. Infection Control Measures

Standard infection control measures are more consistently represented than stewardship programmes and focus on interrupting transmission within neonatal units through hand hygiene, screening, isolation, and environmental cleaning [26,55,56,60,71]. Hand hygiene is the measure most consistently described, with studies identifying strict handwashing, alcohol-based hand rub, and reinforcement of hand hygiene as core elements of both routine prevention and outbreak response [26,42,47,60].

Screening and isolation are predominantly described in outbreak settings or in units with established MDR organism circulation, where rectal swabs, surveillance cultures, and cohorting of colonized infants were used to detect carriage and limit spread [26,38,40,43]. Environmental decontamination is also recurrently reported, particularly in studies implicating equipment, surfaces, incubators, and nursery objects as potential reservoirs of transmission [42,43,44,58,60]. In practice, hand hygiene remains the most consistently reported measure, whereas screening, isolation and environmental cleaning appear especially relevant in high-risk or outbreak contexts.

### 10.3. Microbiome-Based Preventive Strategies

Microbiome-based preventive strategies are only briefly represented in the reviewed studies. The clearest example is the probiotic protocol reported by Kuwelker et al. [70], which proposed the use of defined probiotic strains to reduce colonization with ESBL-producing Enterobacteriaceae and associated adverse outcomes. At present, probiotics are discussed as a preventive approach in this setting.

### 10.4. One Health and Planetary Health Perspectives

The broader ecological dimensions of prevention are less prominently represented than bedside infection prevention and control measures. The most consistent support pertains to environmental reservoirs within healthcare settings, including surfaces, equipment, nursery objects, and the broader NICU environment [42,43,45,60,64]. These findings support the inclusion of environmental control within the overall prevention framework.

Evidence for animal-to-human transmission, by contrast, remains largely conceptual rather than directly demonstrated in neonatal cohorts [77,82]. Evidence for wastewater-related transmission is similarly limited within the reviewed neonatal literature [11,30,41].

A prevention hierarchy can be inferred from the available studies. Standard infection control measures, especially hand hygiene, targeted screening, isolation protocols, and environmental cleaning, are the most consistently supported and immediately applicable strategies [26,42,60]. Antimicrobial stewardship is similarly supported, particularly when local surveillance data inform empirical prescribing decisions [21,35,64].

## 11. Long-Term Outcomes and Public Health Implications

The existing literature on multidrug-resistant (MDR) neonatal sepsis focuses predominantly on acute, in-hospital outcomes, whereas long-term follow-up remains limited [22,24,47,49,71]. As a result, the immediate clinical consequences of these infections are far better documented than their longer-term developmental and neurological impacts.

In the short term, MDR neonatal sepsis is consistently linked to prolonged hospitalization, greater therapeutic complexity, and increased healthcare resource utilization. Several studies, including those by Fox-Lewis et al. [34], Thatrimontrichai et al. [33], Dedeić-Ljubović et al. [38], Pons et al. [17], and Aradhya et al. [14], highlight the considerable burden placed on neonatal services. Fox-Lewis et al. [34] and Pons et al. [17] further suggest that resistant infections are associated with higher direct costs compared with susceptible infections. Even in the absence of formal economic evaluations, multiple reports describe longer hospital stays and greater need for intensive care, underscoring that the impact of MDR sepsis extends beyond mortality alone [22,72].

In contrast, long-term neurodevelopmental outcomes remain poorly defined. Thatrimontrichai et al. [33] is among the few studies to report neurological sequelae following MDR Gram-negative sepsis, while most other studies did not evaluate long-term outcomes or acknowledged them as limitations without formal assessment. This limited evidence base reflects a substantial gap in current knowledge.

From a broader public health perspective, the implications are considerable. Across studies, resistant neonatal infections are described as a continuing burden on already constrained neonatal care systems, particularly in settings with limited diagnostic capacity, inadequate antimicrobial stewardship, and restricted access to effective therapies [24,35,46,47,55,71]. This burden is reflected not only in prolonged admissions and delayed effective treatment, but also in recurrent outbreaks and limited access to active antimicrobial agents [10,42,51,56,68]. Collectively, these findings indicate that MDR neonatal sepsis is not only a clinical challenge at the individual level but also a systemic public health problem that demands stronger prevention, surveillance, and treatment strategies.

## 12. Future Directions and Research Gaps

An important gap in current research is the absence of randomized, neonatal-specific treatment trials. Several studies note that therapeutic decisions still rely on incomplete neonatal pharmacokinetic, pharmacodynamic, efficacy, and safety evidence, especially for newer or salvage regimens [29,49,79,80]. This is particularly relevant because standard empiric regimens have shown poor coverage in some high-burden settings [10,11,14].

Methodological inconsistencies are another relevant limitation. Much of the available literature consists of single-center, retrospective, and observational studies with small samples and heterogeneous definitions [10,22,32,35,46,55,74]. This limits comparability across studies and may hinder the translation of surveillance findings into empirical treatment policy.

The field has also not yet fully integrated genomic and microbiome-based insights. Several studies point to limited molecular typing, incomplete characterization of resistance mechanisms, and an incomplete understanding of transmission pathways in neonatal units [40,59,63,65,83]. Microbiome-related work remains focused mainly on colonization and probiotic concepts rather than integrated clinical microbiome profiling [11,70,73].

Future research should prioritize neonatal-specific clinical trials, standardized surveillance definitions, and more integrated molecular epidemiology. Prospective studies are needed to evaluate alternative empiric and targeted regimens, define age-specific PK/PD targets, and generate neonatal safety and efficacy data, as emphasized by several authors [29,31,49,79,80]. Broader multicenter surveillance with standardized case definitions and susceptibility reporting would also improve comparability and clinical relevance.

Artificial intelligence and personalized antimicrobial therapy may represent important future directions in this field, particularly as potential approaches to current diagnostic and therapeutic limitations.

## 13. An Integrated Conceptual Model for MDR Neonatal Infections

Based on the literature, MDR neonatal infections do not occur in a vacuum; they arise from the intersection of four domains: a vulnerable host, a developing microbiome, an adaptable pathogen, and a healthcare environment that shapes exposure, transmission, and antibiotic pressure. Within this framework, prematurity, low birth weight, critical illness, invasive devices, prolonged hospitalization and repeated antimicrobial exposure increase the likelihood of progression from colonization to invasive infection [12,16,22,27,41,67].

The microbiome component is based primarily on colonization data rather than direct microbiome profiling. Several studies suggest that colonization frequently precedes invasive disease and that maternal and early hospital exposures may shape this process [38,40,43,70,73]. The pathogen component is dominated by hospital-adapted Gram-negative organisms, particularly *Klebsiella pneumoniae*, *Escherichia coli* and *Acinetobacter baumannii*, which have been consistently associated with ESBL production, carbapenem resistance, clonal spread, and persistence in neonatal units [10,19,26,42,56,63,68].

The environmental component includes NICU exposure, cross-transmission, environmental contamination, antimicrobial use, and broader health-system constraints. Other studies indicate that MDR burden is driven largely by hospital acquisition, especially in late-onset sepsis, and is further amplified in settings where diagnostics, surveillance, infection control, and access to effective therapy are limited [11,20,39,44,49,55,64].

These domains converge across a prevention-diagnosis-treatment continuum. Prevention depends on reducing colonization pressure and transmission through maternal risk reduction, hand hygiene, device stewardship, environmental cleaning and surveillance [35,37,45,66]. Accurate diagnosis requires not only the recognition of sepsis, but also anticipation of antimicrobial resistance through local epidemiology, onset-specific patterns, and microbiological or colonization data, where available [20,26,40,56]. Treatment remains challenging because conventional empirical regimens frequently do not reflect current resistance profiles, especially in high-burden units [10,11,14,25,48].

Taken together, this framework demonstrates that MDR neonatal infection is not an isolated microbiological event, but rather the product of interacting host, microbial, and institutional factors [35,49,71,84]. Its significance lies in demonstrating that effective control requires coordinated action spanning prevention, diagnosis, treatment, surveillance, and policy, rather than reliance on any single intervention.

This review has several limitations that should be acknowledged. The available literature is unevenly distributed across geographic settings, with a substantially denser evidence base derived from NICUs in low- and middle-income countries (LMICs) than from population-based studies conducted in high-income countries, which may introduce geographic bias into the synthesis. Furthermore, a considerable proportion of the included evidence originates from single-center studies, outbreak investigations, or pathogen-specific reports, which inherently limit the broader generalizability of the conclusions drawn. The literature search was confined to PubMed/MEDLINE, and eligibility screening was restricted to titles and abstracts, which may have inadvertently excluded relevant studies. Future reviews could be further strengthened by incorporating additional databases such as Embase, Scopus or Web of Science to improve comprehensiveness and reduce the risk of missing relevant literature. The substantial heterogeneity across the included literature, with respect to study design, clinical setting, and outcome definitions, further precluded a formal comparative assessment of study findings. Finally, because the evidence base was synthesized narratively and organized thematically in accordance with the predefined objectives of the review, the resulting synthesis should be interpreted as a comprehensive integrative clinical overview rather than a formal hierarchical ranking of study quality or comparative assessment of evidence strength.

Despite these limitations, this review provides a clinically relevant integrative synthesis of a fragmented literature base and highlights recurring patterns in pathogen distribution, risk factors, diagnostic constraints, and therapeutic challenges across diverse neonatal care settings.

## 14. Conclusions

Multidrug-resistant bacterial infections represent a major threat in neonatal care, particularly in NICUs and resource-limited settings, where host vulnerability, hospital exposure, and antibiotic pressure converge. Across the reviewed evidence, the burden is predominantly driven by resistant Gram-negative pathogens, especially *Klebsiella pneumoniae*, *Escherichia coli* and *Acinetobacter baumannii*, while MRSA remains relevant in selected settings. The most consistent risk factors are prematurity, low birth weight, prolonged hospitalization, invasive devices, and broad-spectrum antibiotic exposure, whereas the roles of maternal colonization and microbiome-related pathways remain incompletely defined.

This review distinguishes itself from earlier literature by providing an integrated, clinically grounded synthesis across epidemiology, risk factors, diagnostics, treatment, prevention, and health-system determinants, rather than focusing on individual pathogens or isolated aspects of neonatal sepsis. Its central message is that MDR neonatal infection is not only a microbiological problem, but also a systems challenge shaped by gaps in surveillance, diagnostic capacity, infection prevention, stewardship, and access to effective therapy.

For clinicians, the principal implications include the need for empirical therapy informed by local surveillance data, early susceptibility-guided adjustment, and rigorous infection control; for policymakers, priorities include strengthening microbiology capacity, stewardship, surveillance, and equitable access to effective antimicrobials. Future progress will require neonatal-specific clinical trials, harmonized multicenter surveillance, and robust molecular epidemiology to mitigate the ongoing impact of MDR neonatal sepsis on neonatal survival and health equity.

## Figures and Tables

**Table 1:** Dominant pathogen group and MDR burden by onset type and region.

	Africa	South and SE Asia	Latin America	Europe/HIC
Both/Unspecified	*Klebsiella* spp. *Acinetobacter* spp. MDR: Very high	*Klebsiella* spp. *Acinetobacter* spp. MDR: Very high	*Klebsiella* spp. ESBL Enterobacterales. MDR: High	GBS, CoNS, *Klebsiella* spp. MDR: Moderate
LOS	*Klebsiella* spp. *Acinetobacter* spp. MDR: Very high	*Klebsiella* spp. *Acinetobacter* spp. MDR: Very high	ESBL Enterobacterales. *Klebsiella* spp. MDR: High	CoNS *Klebsiella* spp. MDR: Moderate
EOS	*E. coli*, GN organisms MDR: High	GBS, *E. coli*, *Klebsiella* spp. MDR: High	*E. coli*, ESBL producers MDR: Moderate	GBS, *E. coli* (susceptible) MDR: Low

**Table 2:** Diagnostic approaches in MDR neonatal infections: clinical utility, limitations, ability to distinguish MDR from non-MDR infection, feasibility in resource-limited settings, and evidence level in reviewed studies.

Diagnostic Approach	Clinical Role	Key Limitations in Neonatal Context	Distinguishes MDR from Non-MDR?	Feasibility in Resource-Limited Settings
Blood culture + antimicrobial susceptibility testing (AST)	Gold standard for confirming bacteremia and sepsis; guides targeted therapy	48–72 h reporting delay; low sensitivity; reduced yield after antibiotic exposure; restricted neonatal blood volume limits repeated sampling	Yes, with susceptibility testing results	Moderate; requires a functional microbiology laboratory
Clinical scoring and bedside assessment	Primary trigger for initiation of empirical antibiotic therapy	Signs are nonspecific; cannot distinguish MDR from non-MDR infection or non-infectious deterioration	No	High; universally accessible; no laboratory infrastructure required
C-reactive protein (CRP) and procalcitonin (PCT)	Adjunct biomarkers to support suspicion of infection and monitor response	Does not distinguish MDR from susceptible infection; influenced by perinatal factors, prematurity, and asphyxia	No	Moderate; available in most hospital laboratories
Leukocyte indices, platelet count, and lactate	Severity assessment and sepsis screening support	Non-specific; affected by prematurity, perinatal asphyxia, and non-infectious conditions	No	High; low cost and widely available
Multiplex PCR panels	Simultaneous pathogen and resistance gene detection	Limited evidence in routine neonatal care; high cost; requires validated platforms and infrastructure	Potentially yes; detects resistance genes directly	Low; cost and infrastructure barriers restrict use
Molecular resistance characterization (PCR-based)	Confirmation of resistance mechanisms; support for outbreak investigation	Not designed for frontline etiologic diagnosis; requires specialized laboratory and interpretive expertise	Yes, at resistance mechanism level	Low; requires specialized laboratory capacity
Whole-genome/hybrid sequencing (WGS)	High-resolution outbreak investigation; confirmation of unusual organisms; transmission tracing	Not a frontline method; long turnaround time; requires bioinformatics infrastructure and dedicated expertise	Yes; highest resolution available	Very low; restricted to specialized referral centers
External or reference laboratory referral	Confirmatory testing when local diagnostic capacity is absent	Logistical burden; turnaround time delays treatment decisions; dependent on transport and communication systems	Dependent on test requested	Context-dependent; varies by setting and referral networks

## Data Availability

The original contributions presented in this study are included in the article/Supplementary Material. Further inquiries can be directed to the corresponding author.

## Supplementary Materials

The following supporting information can be downloaded at: https://www.mdpi.com/article/10.3390/pathogens15050469/s1, Supplementary Material S1: Search strategy and characteristics of the 80 studies included in the narrative synthesis.
