# Supplementary material for: Neonatal Infections Caused by Multidrug-Resistant Bacteria: An Analysis of Prevalence, Risk Factors, and Therapeutic Implications—A Narrative Review
Source: Pathogens. 2026 Apr 26;15(5):469. doi: 10.3390/pathogens15050469 (PMC13209552; doi:10.3390/pathogens15050469)
Supplement: Supplementary file 1 [file pathogens-15-00469-s001.zip › pathogens-4238974-supplementary.pdf]

Appendix 1 – Search strategy and characteristics of the 80 studies included in the narrative synthesis

Appendix 1 reports the PubMed search string applied for study identification (("Infant, Newborn"[MeSH] OR neonate*[Title/Abstract] OR newborn*[Title/Abstract] OR preterm* [Title/Abstract] OR "low birth weight" [Title/Abstract]) AND ("Sepsis"[MeSH] OR "Bacterial Infections"[MeSH] OR sepsis[Title/Abstract] OR "neonatal sepsis"[Title/Abstract] OR "neonatal infection*"[Title/Abstract]) AND ("Drug Resistance, Bacterial"[MeSH] OR "Antimicrobial Resistance"[MeSH] OR antimicrobial resistance[Title/Abstract] OR multidrug resistant[Title/Abstract] OR MDR[Title/Abstract] OR "antibiotic resistance"[Title/Abstract])) and summarizes the principal characteristics of the 80 articles included in the narrative synthesis.

The initial broad PubMed search retrieved approximately 4,696 records, decreasing to 3,074 after restriction by publication year. Subsequently, a focused search strategy requiring resistance-related terms in article titles identified 145 records, which were title and full text screened, resulting in the inclusion of 80 relevant articles in the final narrative synthesis. Prisma flow diagram regarding the search and selection methods is shown as Figure 2 in the main text.

Appendix 1 also summarizes the principal characteristics of the 80 articles included in the narrative synthesis. For each study, we report the bibliographic source, study design, country or region, clinical setting, population characteristics, primary focus, major pathogens or resistance phenotypes addressed, and the main findings relevant to multidrug-resistant neonatal infection. Because the included literature was methodologically heterogeneous and comprised observational studies, reviews, surveillance reports, and guidance documents, the table is intended to improve transparency and facilitate comparison across studies rather than to provide a quantitative pooled analysis.

**First author**	**Year**	**Title**	**Country or countries studied**	**Income setting**	**Study design**	**Study setting**	**Population details**	**Main objective**	**Reported incidence or prevalence of neonatal sepsis**	**Reported prevalence of MDR infections**	**KEY TAKEAWAY**
Lenglet	2020	Rectal screening displays high negative predictive value for bloodstream infection with (ESBL-producing	Haiti	LMIC	other (retrospective analysis of surveillance /clinical microbiology data)	Hospital-based neonatal care unit	Neonates with suspected sepsis after admission to CRUO NCU, Port-au-Prince, Oct 2014-Jul 2018	Assess concordance of rectal swab and blood culture GNB isolates and NPVs for GNB/ESBL-GNB in suspected neonatal sepsis	Background cites ~3 million cases globally/year with 11-19% mortality; study itself did not estimate incidence	Background cites nearly one-half of neonatal infection bacteria resistant to WHO first-/second-line therapy; study itself did not give cohort MDR prevalence	In a low-resource Haitian neonatal unit with repeated MDR-GNB outbreaks, simultaneous rectal screening had modest isolate concordance but high

		) Gram-negative bacteria in neonates with suspected sepsis in a low-resource setting neonatal care unit									negative predictive value, especially for ESBL-positive GNB, supporting its use to guide antibiotic de-escalation during outbreaks.
Ma	2024	Relevance and antimicrobial resistance profile of Klebsiella pneumoniae in neonatal sepsis	China	mixed?	cohort (retrospective comparative observational study)	Single-center NICU / hospital-based	Infants admitted to NICU in special obstetrics and gynecology hospital in Shanghai, Jan 2020-Jun 2022; mostly premature and low birth weight	Compare infection features, clinical characteristics, and resistance of K. pneumoniae vs non-KP Enterobacteriaceae neonatal sepsis	182 sepsis cases treated during study period; KP accounted for 26.9% of positive blood cultures	77.8% of KP group produced ESBLs	This Shanghai NICU study found that K. pneumoniae accounted for a large share of Enterobacteriaceae neonatal sepsis, occurred mainly in premature low-birth-weight infants with late-onset disease, and showed substantial ESBL-mediated resistance dominated by SHV and TEM genes.
Dawoud	2025	Risk factors of diagnosis of and mortality from late	United Arab Emirates	HIC	case-control	Single tertiary-care NICU	NICU of Tawam Hospital, Al-Ain, UAE; Jan 2015-Feb 2019; median	Identify clinical characteristics, risk factors, outcomes,	Background cites Gulf multicenter LOS incidence	In study, MDRO episodes were 29/172 culture-positive LOS episodes	This UAE case-control study found that MDRO late-onset sepsis was

		onset neonatal sepsis caused by multidrug-resistant organisms - a case-control study at a tertiary Hospital in the UAE					age 24 d; median weight 1042 g; 82% preterm	and resistance patterns for MDRO versus non-MDRO LOS	11.6/1000 live births and 56.1/1000 NICU admissions		dominated by ESBL Enterobacteriaceae, and that female sex, necrotizing enterocolitis, and umbilical arterial catheter use independently predicted MDRO infection, while mortality was similarly high in MDRO and non-MDRO LOS.
Sharland	2019	Safety and Efficacy of Tigecycline to Treat Multidrug-resistant Infections in Pediatrics: An Evidence Synthesis	Mixed / not neonatal-specific	mixed	review	Pediatric clinical trials, expanded access, databases, pharmacovigilance	Children; no neonatal-specific clinical trial cohort reported	Synthesize published and unpublished tigecycline data in children, including PK, safety, and real-world use	Not reported	Not reported	This paper is relevant mainly as a pediatric salvage-therapy review rather than a neonatal MDR sepsis study: it supports tigecycline as a possible option when alternatives are lacking, but provides no neonatal-specific clinical trial data and no direct epidemiology

											of neonatal MDR infection.
Alghamdi	2025	Successful dual therapy of aztreonam and ceftazidime-avibactam for multidrug-resistant Stenotrophomonas maltophilia infection in a preterm neonate: A life-saving approach	Saudi Arabia	HIC	other (case report)	NICU / hospital-based	Preterm male neonate born at 30 weeks + 4 days; birth weight 1135 g; mechanically ventilated	Report successful use of aztreonam plus ceftazidime-avibactam for MDR S. maltophilia infection in a preterm neonate	Not reported	Not reported	This case report shows that dual aztreonam plus ceftazidime-avibactam can rescue a preterm neonate with MDR S. maltophilia respiratory infection when standard options fail, but the evidence remains limited to a single highly complex case.
Cailes	2015	The current and future roles of neonatal infection surveillance programmes in combating antimicrobial resistance	Mixed / review with examples from the UK, Europe and other surveillance networks	mixed	review	NICU / multicenter / hospital-based surveillance	Neonates, especially premature and low birth weight infants; NICU populations	To review the current and future roles of neonatal infection surveillance programmes in combating antimicrobial resistance.	EOS in the UK reported as 0.5-0.9 per 1000 live births and 9 per 1000 neonatal admissions; LOS/HABSI cumulative incidence reported	No single pooled MDR prevalence reported.	This review shows that MDR neonatal infection is strongly linked to surveillance quality, NICU practice and preventable hospital-acquired bloodstream infection. It emphasizes that preterm and low birth

									as 3-23% and 2-15 per 1000 patient-days; CLABSI 1-20% and 3-22 per 1000 catheter-days.		weight infants are especially vulnerable, that Gram-negative burden varies by region, and that benchmarking and stewardship depend on harmonized surveillance systems.
Ağın	2011	The evaluation of clusters of hospital infections due to multidrug-resistant Salmonella enterica serovar typhimurium in the neonatal unit: a two-year experience	Turkey	LMIC	outbreak investigation	NICU / hospital-based	37 premature and 44 term neonates in a neonatal unit with clustered MDR Salmonella infection.	To evaluate clustered hospital infections due to MDR Salmonella enterica serovar Typhimurium in a neonatal unit over two years.	Not population-based; seven clusters documented between April 2002 and March 2004.	All outbreak isolates were ESBL-producing MDR Salmonella; article notes prior Turkish report of 76% multidrug resistance among S. typhimurium isolates.	This study directly demonstrates that clonal ESBL-producing MDR Salmonella can produce repeated NICU clusters with mixed colonization, gastroenteritis and invasive infection. It shows how overcrowding, understaffing and environmental contamination sustain transmission, and how active surveillance plus stronger

											infection-control measures can stop outbreaks.
Nakwan	2019	The Use of Colistin for the Treatment of Multidrug-resistant Gram-negative Infections in Neonates and Infants: A Review of the Literature	Mixed / multiple countries across included studies	mixed	review	NICU / hospital-based	Mostly preterm, critically ill neonates and infants treated with colistin for MDR Gram-negative infection.	To review the efficacy, safety and pharmacokinetics of colistin for MDR Gram-negative infections in neonates and infants.	Not reported as a pooled incidence.	No pooled prevalence; review states MDR Gram-negative infections are increasing globally and are more common in lower-middle-income countries.	This review shows how restricted therapy becomes once MDR Gram-negative neonatal infection is established. Colistin appears to provide useful salvage activity against invasive infections caused especially by Acinetobacter, Klebsiella and Pseudomonas, but neonatal pharmacokinetics, nephrotoxicity and other toxicities remain important unresolved issues.
Kuwelker	2021	Use of probiotics to reduce infections and death	Tanzania	LMIC	clinical trial	hospital-based plus community follow-up	Healthy term newborn infants weighing at least 2.0 kg,	To evaluate whether probiotics reduce death /	No study results yet; background notes	Background reports around two-thirds of Tanzanian infants below 3 months had	This protocol is highly relevant for the prevention and

		and prevent colonization with extended-spectrum beta-lactamase (ESBL)-producing bacteria among newborn infants in Tanzania (ProRIDE Trial): study protocol for a randomized controlled clinical trial					enrolled within the first 3 days of life and followed to 6 months of age.	hospitalization and ESBL-producing Enterobacteriaceae colonization in Tanzanian newborns.	infection as a major contributor to infant mortality.	fecal carriage of ESBL-E.	microbiome sections of an MDR neonatal infection review. It frames early-life ESBL colonization as a modifiable precursor to severe infection and proposes probiotics as a practical strategy to strengthen colonization resistance in a low-resource African setting.
Sáez-López	2016	Vaginal versus Obstetric Infection Escherichia coli Isolates among Pregnant Women: Antimicrobial Resistance and Genetic Virulence Profile	Spain	HIC	other	hospital-based	Pregnant women with vaginal colonization or obstetric infection; isolates from vaginal samples, endometrial aspirate, placental and amniotic fluid samples.	To compare antimicrobial resistance and genetic virulence profiles of vaginal versus obstetric infection E. coli isolates among pregnant women.	Not a neonatal sepsis cohort.	No formal MDR prevalence reported; 65.5% of isolates were resistant to ampicillin and only one isolate had ESBL (CTX-M-15).	This paper is most useful for the maternal and vertical-transmission side of an MDR neonatal infection review. It shows that maternal vaginal E. coli colonization and obstetric infection share virulent traits, that ampicillin

											resistance is common, and that pPROM is an important associated risk factor, raising concern about standard empiric regimens in mothers and newborns.
Williams	2022	Antibiotics needed to treat multidrug-resistant infections in neonates	Global review with emphasis on LMIC settings; examples include India, Bangladesh, Pakistan and multicountry cohorts	mixed	review / policy paper	global literature and policy review	Neonates with suspected or confirmed sepsis, especially those at risk of MDR hospital-acquired gram-negative infection	Re-evaluate priority neonatal MDR pathogens and propose a prioritized antibiotic development agenda for neonates.	Cites ~3 million cases of neonatal sepsis annually and up to 570,000 sepsis-attributable deaths globally.	Notes MDR gram-negative pathogens exceed 80% of causative pathogens in some LMIC neonatal settings.	This paper frames MDR neonatal infection as a global access-and-evidence problem rather than a single-center epidemiology issue. It shows that MDR gram-negative sepsis, especially due to Klebsiella and Acinetobacter, is driving excess mortality in neonates, particularly in LMICs. At the same time, very few new antibiotics have neonatal labeling or trial data. The

											main contribution is a call for prioritized, harmonized neonatal antibiotic development and regulatory acceleration.
Freitas	2024	Antimicrobial resistance and epidemic clustering of late-onset neonatal infections in a Brazilian intensive care unit	Brazil	LMIC	retrospective cohort / outbreak investigation	single tertiary referral NICU, hospital-based	Neonates with blood-culture-confirmed LOS admitted 2012-2016 and 2018-2021; median birth weight 1355 g, median gestation 31 weeks; 55% <1500 g	Describe LOS pathogens, resistance profiles, epidemic microclusters, and mortality association of MDR infection in a Brazilian NICU.	Does not report incidence per live births; all data are blood-culture-confirmed LOS episodes.	A third of Enterobacterales were resistant to cefepime; a third of non-fermenters resistant to carbapenems. MDR exposure associated with death.	This study shows that MDR LOS in a Brazilian NICU clusters frequently in time, consistent with cross-transmission. The clearest example was a carbapenem-resistant A. baumannii outbreak in which rectal colonization and bloodstream isolates shared blaOXA-23 and a single RAPD clone. Importantly, MDR infection was independently associated with higher odds of death. The paper is

											especially useful for linking bedside epidemiology, colonization data, and outbreak molecular typing in an LMIC NICU.
Williams	2024	Antimicrobial resistance in Enterobacterales, Acinetobacter spp. and Pseudomonas aeruginosa isolates from bloodstream infections in Australian children, 2013-2021	Australia	HIC	surveillance	national multicenter hospital surveillance including tertiary pediatric hospitals and level III NICUs	Hospitalized children <18 years, including neonates, from 26 hospitals in 2013 increasing to 35 by 2021	Describe epidemiology, AMR trends, and outcomes of Enterobacterales, P. aeruginosa and Acinetobacter BSIs in Australian children over time.	Highest bacteremia incidence noted in neonates, but neonatal-specific sepsis prevalence not separately quantified in excerpt.	MDR defined as resistance in >=3 classes; MDR associated with higher odds of death; more frequent in older children than neonates.	This national Australian surveillance study is valuable as a high-income comparator showing that pediatric gram-negative resistance is rising over time, especially in Enterobacterales, while carbapenem resistance remains uncommon. Neonates account for a substantial share of bloodstream infections, but the analysis is not neonatal-only. Its main contribution to an MDR neonatal

											review is contextual: it demonstrates that even in HICs, AMR trends require pediatric-specific surveillance and are linked to worse outcomes.
Fox-Lewis	2018	Antimicrobial Resistance in Invasive Bacterial Infections in Hospitalized Children, Cambodia, 2007-2016	Cambodia	LMIC	surveillance / retrospective cohort	single hospital-based pediatric surveillance site	Hospitalized children at Angkor Hospital for Children; all births occurred outside the hospital; neonatal subgroup analyzed	Describe 10-year AMR trends, mortality, and cost burden of invasive bacterial infections in hospitalized children, with neonatal age comparisons.	Not a birth cohort; no neonatal sepsis incidence per live births reported.	MDR in K. pneumoniae 81.8%, E. coli 82.1%, A. baumannii 93.3%.	This Cambodian surveillance study shows how severe MDR burden can be in a low-resource pediatric setting, with especially high resistance in neonatal Klebsiella isolates and extremely high MDR rates in K. pneumoniae, E. coli, and A. baumannii. It also goes beyond microbiology by linking resistance to mortality, ICU admission, and higher costs. For a

											neonatal MDR review, its main value is demonstrating how health-system resource constraints and hospital acquisition amplify the consequences of resistance.
Cailes	Not stated in snippet	Antimicrobial Resistance in UK Neonatal Units: neonIN Infection Surveillance Network	United Kingdom	HIC	retrospective analysis of prospectively collected surveillance data	30 neonatal units; multicenter, hospital-based	Newborns admitted to participating units with positive blood, CSF or sterile urine culture treated for at least 5 days	Define susceptibilities of common neonatal sepsis pathogens in the UK and inform empiric antibiotic guidance.	Not reported in the extracted snippet.	Does not primarily report MDR prevalence; focuses on coverage of empiric combinations and resistance differences between EOS and LOS.	The UK neonIN analysis is useful because it separates EOS from LOS and shows that resistance patterns differ meaningfully by timing of onset. Standard EOS regimens still covered most pathogens, but LOS isolates were more resistant, supporting distinct empiric strategies for hospital-acquired disease. The paper contributes more to

											stewardship and regimen selection than to molecular MDR epidemiology. In a comparative review, it serves as a high-income surveillance benchmark where resistance exists but remains more manageable than in many LMIC NICUs.
Guiral	2012	Antimicrobial Resistance of Escherichia coli Strains Causing Neonatal Sepsis between 1998 and 2008	Spain	HIC	Retrospective laboratory-based observational study / surveillance of isolates	Single tertiary hospital; EONS and hospital-acquired LONS	Neonates with E. coli sepsis at Hospital Clinic of Barcelona, 1995-2008; 78% of EONS isolates from premature neonates; EONS cases associated with prolonged membrane rupture and/or chorioamnionitis	Evaluate antimicrobial resistance of E. coli strains causing EONS and LONS and their temporal evolution	Not directly reported for the cohort; background cites rising E. coli EOS in VLBW infants	Not explicitly quantified as MDR rate; resistance across multiple classes increased over time	In this Spanish single-center series, E. coli causing both EONS and hospital-acquired LONS showed rising resistance over time, especially to gentamicin, piperacillin, and tobramycin. ESBL determinants (CTX-M-14 and CTX-M-15) and class 1 integrons

											were already present, indicating mobile resistance elements in neonatal sepsis isolates. Prematurity and perinatal risk factors characterized many EONS cases. The paper mainly supports stronger AMR surveillance and reconsideration of ampicillin/gentamicin-based empiric therapy.
Cole	2012	Antimicrobial resistance, infection control and planning for pandemics: The importance of knowledge transfer in healthcare resilience and	Primarily UK / EU / US policy context	HIC	Narrative policy review / commentary	Healthcare systems and emergency planning; not neonatal-specific clinical cohort	Healthcare systems, hospitals, policy and resilience planning; neonatal care referenced as one example of antibiotic overuse	Explore cross-disciplinary AMR policy challenges and links between infection control, healthcare resilience, and pandemic planning	NR	NR for neonatal MDR specifically	This article is not a neonatal sepsis cohort, but it is highly relevant for stewardship context. It argues that antibiotic overuse in neonatal care is substantial, citing UK guidance that many newborns treated for suspected

		emergency planning									EOS are ultimately uninfected. The paper links AMR control to health-system resilience, emphasizing standardized protocols, better diagnostics, and data systems. For a narrative review, it is best used as a policy/stewardship background source rather than as direct neonatal MDR microbiology evidence.
Uppal	2025	Bacterial infections and antimicrobial resistance patterns: a comprehensive analysis of health dynamics across regions in Pakistan (2013-2023)	Pakistan	LMIC	Retrospective laboratory surveillance / cross-sectional analysis	Laboratory-based multicity/regional blood and urine sample analysis; not NICU-specific	Patients of multiple ages in Pakistan; infants/neonates analyzed as age subgroup in blood cultures	Describe pathogen prevalence, demographic patterns, and AMR trends in blood and urine samples across Pakistani regions over time	Not a dedicated neonatal sepsis incidence study; infants/neonates were 13.0% of blood cases in 2013-2017 and 16.76% in 2017-2020	No neonatal-specific MDR prevalence; discusses AMR/XDR burden generally	This Pakistan study offers broad regional AMR context rather than a dedicated neonatal MDR sepsis analysis. It shows that infants/neonates remain an important subgroup in bloodstream infections, while health-

											system problems such as antibiotic misuse, weak regulation, and regional disparities likely sustain AMR pressure. The paper is useful for contextual LMIC epidemiology and stewardship arguments, but it provides limited neonatal-specific MDR, EOS/LOS, or NICU detail. It should therefore be used as background epidemiology rather than core neonatal MDR evidence.
Darlow	2022	Flomoxef for neonates: extending options for treatment of neonatal sepsis caused by	Not a clinical cohort; pharmacometric response from UK authors using public-domain	Mixed / not directly applicable	Authors' response / pharmacokinetic modeling commentary	Population pharmacokinetic model for neonates aged <28 days; not an interventional clinical trial in NICU	Neonates <4 weeks; modeled infants predominantly aged <28 days; simulations include older/heavier infants	Defend and clarify a population PK model supporting flomoxef dosing options for neonatal sepsis caused by	NR	Targets ESBL-producing Enterobacterales conceptually; no prevalence estimate in uploaded response	The uploaded Darlow document is an authors' response defending a neonatal flomoxef population PK model, not a primary

		ESBL-producing Enterobacterales-authors' response	neonatal PK data				within neonatal range	ESBL-producing Enterobacterales			clinical outcomes paper. Its main value for a narrative review is therapeutic: flomoxef may be a useful option against ESBL-producing Enterobacterales in neonates, but dosing relies on age-restricted PK modeling and still needs cautious interpretation. It contributes PK/PD and developmental pharmacology insight rather than epidemiology or NICU transmission data. Any conclusions should therefore be explicitly limited to model-based treatment support.
Wang	2020	Literature review on the	China	Mixed upper-middle /	Literature review of retrospectiv	Hospital-based; many NICU-related	Chinese neonates with	Summarize bacterial distribution	Not pooled incidence;	Not given as single pooled MDR rate; MRSA and MRCoNS	This review provides strong

		distribution characteristics and antimicrobial resistance of bacterial pathogens in neonatal sepsis		not categorized in paper; treated here as mixed-middle income context	e hospital studies	studies from 24 cities in 14 Chinese provinces	sepsis/bloodstream infection; literature from 2016 to April 2018	and antimicrobial resistance patterns in Chinese neonatal sepsis to inform empirical therapy	review notes positive blood culture rates in some developing countries can reach 40-50%	detection rates exceeded national average; K. pneumoniae resistance to third-generation cephalosporins exceeded national average	descriptive evidence that Chinese neonatal sepsis is dominated by CoNS among Gram-positive isolates and by Klebsiella and E. coli among Gram-negative isolates. Resistance patterns suggest serious concern for MRSA/MRCoNS and cephalosporin-resistant K. pneumoniae, while carbapenems and selected aminoglycosides retained activity against many Gram-negative enteric pathogens. Its value lies in broad pathogen distribution and empirical-therapy guidance, though it lacks

											mechanistic, genomic, microbiome, and outcomes data. It is a core background source for NICU-associated pathogen ecology and stewardship in China.
Pérez-Viso	2024	A long-term survey of Serratia spp. bloodstream infections revealed an increase of antimicrobial resistance involving adult population	Spain	HIC	Retrospective hospital surveillance/genomic epidemiology study	Hospital-based; adult wards/ICUs; no NICU	Mostly adults/elderly; only 6 patients aged 0-19 years; not a neonatal cohort	To assess incidence, clinical/microbiological features, and AMR evolution of Serratia bloodstream infections over 2005-2020	Not a neonatal sepsis study	Increase over time; ESBL genes in 3 isolates, blaVIM-1 carbapenemase in 7 isolates, one with both	This is not a neonatal study, but it is mechanistically relevant because it shows Serratia can acquire ESBL and carbapenemase determinants and spread nosocomially via plasmids. The study used WGS to reveal blaVIM-1, blaSHV-12, IncL/M plasmids, and multiple genotypes in hospital BSI isolates. For a neonatal MDR review, it serves as

											contextual evidence on Serratia's resistance ecology rather than direct neonatal epidemiology.
Yeshiwas	2025	A mixed-method study on antimicrobial resistance infection drivers in neonatal intensive care units: pathways, risks, and solutions	Ethiopia	LMIC	Mixed-methods cross-sectional study	Single-center NICU; clinical + environmental sampling	NICU neonates at Felege Hiwot Hospital; many admitted for LBW, prematurity, RDS, jaundice	To evaluate AMR prevalence, risk factors, outcomes, and infection control measures in a NICU	35% samples positive for AMR pathogens; sepsis among admission causes collectively 10%	Very high resistance among major pathogens; Gentamicin/Cotrimoxazole/Ciprofloxacin resistance 98-100%	This NICU study links MDR burden directly to modifiable infection-control failures rather than pathogen prevalence alone. CONS, Klebsiella pneumoniae, and Acinetobacter spp. showed near-complete resistance to several commonly used agents, while amikacin retained relative activity. Environmental contamination of incubators and infrastructure failures such as non-functional

											sinks, poor PPE use, and inadequate disinfection emerged as major drivers of transmission.
Viswanathan	2011	Aetiology and Antimicrobial Resistance of Neonatal Sepsis at a Tertiary Care Centre in Eastern India: A 3 Year Study	India	LMIC	Retrospective review/surveillance of culture-positive sepsis	Single-center Level III NICU; hospital-based	Inborn and outborn NICU neonates, many LBW/VLBW and high-risk maternal background	To review etiologic agents of neonatal sepsis and their antibiotic resistance pattern over 3 years	14.8/1,000 live births among inborn babies; 8.3% among outborn NICU admissions	No composite MDR prevalence; high resistance to ampicillin 98.5%, gentamicin 84.4%, amikacin 65.6%, cefotaxime 81.3%	This study shows classic Gram-negative predominance in an Indian NICU, with Klebsiella pneumoniae leading and Acinetobacter emerging as a serious secondary pathogen. Resistance to standard WHO-aligned first-line regimens was extremely high, prompting a change in empirical treatment policy. The similarity between EOS and LOS etiology suggests early horizontal transmission and persistent

											nosocomial pressure.
Okomo	2019	Aetiology of invasive bacterial infection and antimicrobial resistance in neonates in sub-Saharan Africa: a systematic review and meta-analysis in line with the STROBE-NI reporting guidelines	26 countries in sub-Saharan Africa	Predominantly LMIC	Systematic review and meta-analysis	Mostly hospital-based; one community study	Neonates with microbiologically confirmed invasive bacterial infection including sepsis/bacteremia and meningitis	To synthesize causes of invasive bacterial infection and AMR in neonates in sub-Saharan Africa and assess reporting quality	Not a pooled incidence estimate; burden described as major and hospital-acquired infections common	Resistance to WHO β-lactams 68% (614/904); aminoglycosides 27% (317/1176)	This meta-analysis establishes that neonatal invasive bacterial infection and AMR in sub-Saharan Africa are substantial, heterogeneous, and often poorly documented. S. aureus, Klebsiella spp., and E. coli dominate reported sepsis/bacteremia, while resistance to WHO-recommended β-lactams is strikingly high. The paper's major contribution is not only epidemiologic synthesis, but also the demonstration that weak reporting and limited microbiology infrastructure are major

											barriers to understanding MDR neonatal infection across the region.
Jajoo	2018	Alarming rates of antimicrobial resistance and fungal sepsis in outborn neonates in North India	India	LMIC	Prospective observational cohort study	Single outborn level-3 neonatal unit within multicenter DeNIS collaboration; hospital-based	Outborn neonates admitted to tertiary unit; many ≥32 weeks and ≥1500 g; substantial prior healthcare exposure; 20% home born	To evaluate sepsis prevalence/outcomes and characterize pathogen profile and AMR among outborn neonates	Culture-positive sepsis 13.1%; total sepsis 54.7%	Among key Gram-negatives: K. pneumoniae MDR 78.0%, A. baumannii MDR 91.3%	This study captures a particularly vulnerable group-outborn neonates with mixed community and healthcare exposures-and shows a very high burden of sepsis, death, fungal infection, and MDR Gram-negative disease. Klebsiella pneumoniae and Acinetobacter baumannii were both common and frequently carbapenem-resistant, while prior antibiotic exposure before referral was widespread. The paper is

											especially valuable because it shows MDR neonatal sepsis is not confined to extremely premature infants, but also affects larger and more mature outborn babies.
Angulo-Zamudio	2025	Characteristics of Neonates with Sepsis Associated with Antimicrobial Resistance and Mortality in a Tertiary Hospital in Mexico	Mexico	LMIC	Retrospective observational study	Hospital-based neonatal unit / tertiary hospital	Neonates ≤28 days with signs/symptoms of sepsis and positive blood cultures; August 2021-April 2023	Describe epidemiologic, clinical, laboratory and microbiologic characteristics of neonatal sepsis; compare EOS vs LOS; assess AMR and mortality associations	4.0% (314/8,382)	52.6% MDR; 10.8% XDR; 5.5% PDR	This is the most directly informative clinical paper in the uploaded set. It shows that late-onset neonatal sepsis predominated in this Mexican tertiary setting, and over half of isolates were multidrug resistant. Prematurity, low birth weight, cesarean delivery, and mechanical ventilation were strongly linked to

											resistant infections, while several clinical and laboratory markers tracked with mortality. Overall, the study frames MDR neonatal sepsis as a high-burden NICU-linked syndrome that requires earlier diagnosis and better local resistance surveillance.
González -Nava	202 4	Antibacter ial activity of Nocardia spp. and Streptomy ces sp. on multidrug -resistant pathogens causing neonatal sepsis	Mexico	LMIC	Experiment al in vitro laboratory study	Laboratory-based study using MDR neonatal pathogens from ICU/NICU isolates	Actinobacteri a from clinical actinomyceto ma cases tested against MDR pathogens implicated in neonatal sepsis/pneu monia	Detect biosynthetic gene clusters and evaluate antimicrobial activity of actinobacteri al strains against MDR pathogens causing neonatal sepsis	Not measured ; backgrou nd cites neonatal sepsis burden in LMICs	Not measured as cohort prevalence; all 5 target pathogens were MDR	This paper is not a clinical epidemiology study, but it is highly relevant for the therapeutics section of an MDR neonatal sepsis review. It tested actinobacterial strains against real MDR neonatal pathogens, including ESBL-producing organisms, and found

											broad inhibitory activity, especially for Streptomyces albus 94.1572. The work supports the search for new anti-infective compounds when standard neonatal regimens lose activity. Its main value lies in therapeutic innovation rather than burden estimation or risk-factor analysis.
Butin	2017	Chromogenic detection procedure for the multidrug-resistant, neonatal sepsis-associated clone Staphylococcus capitis NRCS-A	France; validation used isolates from four countries and samples from a French NICU setting	HIC / mixed	Diagnostic validation / surveillance-method note	NICU-linked laboratory, biological, and environmental surveillance samples	Neonatal stools, neonatal skin, pregnant women vaginal samples, and NICU environmental samples	Validate a practical chromogenic screening method to detect the MDR neonatal sepsis-associated Staphylococcus capitis NRCS-A clone	Not measured	Not measured as clinical prevalence; focuses on an endemic MDR clone	This paper is especially valuable for the NICU transmission and screening sections of the review. It shows that the globally disseminated Staphylococcus capitis NRCS-A clone can be detected in biological and environmental

											NICU samples using a practical chromogenic approach. The study supports the idea that MDR neonatal sepsis is partly sustained by colonization reservoirs and persistent clone circulation, not only by treatment failure. Its main contribution is to infection control and surveillance rather than clinical outcomes.
Bekele Sharew	2025	Bacterial profile and antimicrobial resistance patterns among pediatrics patients with suspected bloodstream infections in	Ethiopia	LMIC	Systematic review and meta-analysis of laboratory-based observational studies	National synthesis of pediatric hospital and NICU studies; mixed wards	Pediatric patients (≤18 years) with suspected bloodstream infection; subgroup analyses included neonates and NICU patients	Estimate pooled prevalence of culture-confirmed pediatric bloodstream infection and pooled antimicrobial resistance/MDR patterns in Ethiopia	Overall pooled pediatric BSI prevalence 30.66% (95% CI 27.18-34.15); neonates 34.07%; NICU patients 35.83%	Overall pooled MDR prevalence 80.54% (95% CI 77.24-83.85); NICU subgroup 78.35%; neonates 78.86%	This review shows that pediatric bloodstream infections in Ethiopia are common and highly resistant, with especially severe MDR burden among Gram-negative organisms. Klebsiella spp.

		Ethiopia: a systematic review and meta-analysis									dominated the bacterial profile and, together with Acinetobacter spp., reached extreme pooled MDR levels. Neonates and NICU patients appeared especially affected. The study strongly supports urgent investment in diagnostics, routine susceptibility testing, surveillance, infection prevention and antimicrobial stewardship.
T. Naas	2016	Neonatal infections with multidrug-resistant ESBL-producing E. cloacae and K. pneumoniae in Neonatal Units of two different	Madagascar	LMIC	Two-hospital neonatal-unit molecular epidemiology study / outbreak investigation	Hospital-based neonatal units in two Antananarivo hospitals	Newborns with suspected early neonatal infection (day 0 or 2 samples); mean gestational age 38±3.5 weeks; 60/303 preterm (<38 weeks)	Characterize molecular mechanisms of β-lactam resistance and epidemiology of ESBL-producing neonatal Enterobacterales in two Malagasy neonatal units	Among 303 suspected early neonatal infection cases, 39 newborns had ESBL-producing E. cloacae and/or K. pneumon	All but 4 studied ESBL isolates were MDR; all ESBL-producing isolates analyzed were described as multidrug resistant in detailed antibiograms	This study documents severe early neonatal infection caused by ESBL-producing E. cloacae and K. pneumoniae in two Malagasy neonatal units. CTX-M-15/TEM-1-

		Hospitals in Antananarivo, Madagascar							iae; among 254 blood cultures, 76 proven infections remained after excluding contaminants		carrying plasmids and multiclonal spread showed that resistance was not confined to a single clone, and both community carriage and very early nosocomial contamination likely contributed. Because carbapenems were unavailable, affected newborns were treated with broader cephalosporins and mortality was very high. The paper strongly highlights how molecular surveillance can reveal hidden transmission networks in low-resource neonatal care.
Angela Dramowski	2015	Neonatal nosocomial	South Africa	LMIC	Retrospective surveillance	Referral hospital neonatal	Hospitalized neonates at Tygerberg	Determine burden, pathogen	Nosocomial BSI rate	High resistant-pathogen prevalence: MRSA 66%, MDR A.	This study shows that nosocomial

		bloodstream infections at a referral hospital in a middle-income country: burden, pathogens, antimicrobial resistance and mortality			/ record review	service with NICU, high-care and special-care wards	Hospital, Cape Town, 2009-2013; service cared for sick and/or low-birthweight infants in a 128-bed neonatal unit	profile, antimicrobial resistance and mortality of nosocomial neonatal bloodstream infection	3.9/1,000 patient days (95% CI 3.6-4.2), unchanged over time	baumannii 90%, ESBL K. pneumoniae 73%, MDR K. pneumoniae 71%	neonatal bloodstream infection remained a major problem in a large South African referral neonatal service over five years. Gram-negative organisms dominated, especially K. pneumoniae, and major resistant phenotypes such as ESBL K. pneumoniae, MDR A. baumannii and MRSA were common. Mortality was substantial and concentrated in very low birth weight infants. The findings point to the need for stronger surveillance, better sampling practice and

											focused infection prevention in the NICU.
Josephine Tumuhamye	2020	Neonatal sepsis at Mulago national referral hospital in Uganda: Etiology, antimicrobial resistance, associated factors and case fatality risk	Uganda	LMIC	Hospital-based cross-sectional study	Tertiary referral hospital pediatric emergency care unit	Neonates <29 days presenting with clinical signs of sepsis at Mulago national referral hospital, Kampala, Jan-Dec 2018	Determine culture positivity, bacterial etiology, antimicrobial resistance, factors associated with culture-positive sepsis and case fatality	46/359 (12.8%; 95% CI 9.5-16.7) had culture-positive sepsis	ESBL producers 6.5% (3/46 isolates); MRSA in 19/359 neonates and about one-third of S. aureus isolates; 73.9% of isolates resistant to ampicillin and 23.9% to gentamicin	At Mulago referral hospital, only about one in eight neonates with clinical sepsis had a culture-confirmed pathogen, but resistance among those pathogens was clinically important. S. aureus predominated and many isolates were MRSA, while a smaller subset of Gram-negative isolates showed ESBL resistance. Cesarean delivery was the clearest measured risk factor for culture-positive sepsis, and standard first-line therapy did not reliably cover

											all isolates. The study argues that local treatment guidelines should be updated using current resistance data.
Dustin D. Flannery	2022	Neonatal multidrug resistant gram-negative infection: epidemiology, mechanisms of resistance, and management	United States and international literature	Mixed	Narrative review	NICU-focused review of single-center studies, collaborative reports and surveillance datasets	Hospitalized neonates, especially preterm NICU infants, with focus on MDR Gram-negative colonization and infection	Review epidemiology, resistance mechanisms, management, prevention and knowledge gaps for neonatal MDR Gram-negative infection, especially ESBL-producing Enterobacterales and CRE	Narrative review; no single pooled incidence. Notes that Gram-negatives cause ~15-30% of LOI in the U.S., >50% of LOI in some Chinese/ Brazilian cohorts, ~40% of neonatal infections in sub-Saharan Africa and two-thirds in India	Variable across studies. Review notes 50-80% neonatal colonization with MDR-GN in reports from Asia/South America/Africa; ESBL colonization >50% in one Ecuadorian NICU; CRE colonization 5-9% in India/Cambodia; neonatal ESBL/CRE remain rare in the U.S.	This review synthesizes why MDR Gram-negative infection has become one of the most important threats in contemporary neonatal care. ESBL-producing Enterobacterales and CRE are especially problematic in preterm NICU infants because colonization is common, transmission is efficient, and evidence for treatment in neonates remains limited. The review makes clear that

											prematurity, prolonged hospitalization, invasive devices and antibiotic exposure drive risk, while local antibiograms and strong infection prevention are essential for management. It also highlights the urgent need for neonatal-specific data on newer antibiotics and better global surveillance.
Mulatu Gashaw	2024	Neonatal Sepsis Due to Multidrug-resistant Bacteria at a Tertiary Teaching Hospital in Ethiopia	Ethiopia	LMIC	Observational longitudinal study	Single tertiary-hospital NICU (Jimma Medical Center)	Neonates admitted to NICU with presumptive sepsis; one blood sample (1-3 mL) and CSF when meningitis suspected	Determine bacterial etiologies, MDR burden and resistance patterns among neonates with sepsis in Jimma, Ethiopia	Culture identified pathogens in 152 isolates from 238 presumptive sepsis cases; authors report 63.5% culture positivity	Very high MDR burden; 74.4% of Gram-negatives were ESBL producers and 57.1% of S. aureus were MRSA	This Ethiopian NICU study shows a very high burden of MDR neonatal sepsis, with ESBL-producing Gram-negatives and MRSA common. S. aureus, K. pneumoniae and E. coli predominated, while A. baumannii

											showed notable carbapenem resistance. Standard first-line antibiotics performed poorly, and two-thirds of deaths were linked to MDR pathogens. The study underscores how limited diagnostics and antibiotic access amplify mortality risk in low-resource neonatal care.
Chloé Guitart	2020	Neonates, Sepsis, and Antimicrobial Resistance-Steps to Tackle Difficult Issues in Our Most Vulnerable Population	Not country-specific; global perspective from Switzerland/Iran authorship	Mixed	Editorial / commentary	Global maternal-newborn and hospital infection prevention context	Hospitalized neonates and maternal-newborn dyads discussed conceptually	Highlight the burden of neonatal sepsis and antimicrobial resistance and advocate hand hygiene and stewardship	Cites 50 million sepsis cases and 11 million sepsis-related deaths in 2017 globally; more than half among children, mostly neonates	No original estimate; notes AMR may account for ~30% of neonatal sepsis deaths, mostly in LMICs	This editorial frames neonatal sepsis and AMR as interconnected global patient-safety problems. It distinguishes EOS as primarily vertical transmission and LOS as largely horizontal transmission from hands, devices,

											community contacts and nutritional sources. The authors argue that hand hygiene remains the single most important preventive intervention, supported by stewardship to avoid resistance driven by antibiotic overuse. The piece is most useful for the narrative review's health-systems and infection-prevention framing rather than for microbiologic detail.
Annette Onken	2015	Prevalence and Antimicrobial Resistance of Microbes Causing Bloodstream Infections	Tanzania (Unguja, Zanzibar)	LMIC	Prospective cohort / surveillance study	Referral and teaching hospital; medical, pediatric and neonatal departments	Consecutively enrolled neonates, children and adults with signs of systemic infection at Mnazi Mmoja Hospital	Describe pathogens causing bloodstream infection and their antimicrobial susceptibility in Zanzibar	Not neonatal-sepsis specific; pathogenic microbes isolated from 14.0% (66/470) blood	5 ESBL Enterobacteriaceae BSIs overall; 6/7 Salmonella Typhi isolates were MDR; neonatal-specific MDR prevalence not separately reported	This Zanzibar study is not neonatal-only, but it is important for contextualizing bloodstream infection epidemiology in East Africa. K. pneumoniae,

		in Unguja, Zanzibar							cultures overall		E. coli, Acinetobacter spp. and S. aureus predominated, and the detection of community-acquired ESBL bloodstream infection was especially concerning. Because the study included 113 neonates within a broader cohort, it offers limited neonatal-specific MDR detail, but it clearly shows that difficult-to-treat Gram-negative resistance had already spread beyond hospitals. For a narrative review, it is most informative as a regional epidemiology and stewardship signal rather than a NICU-

											focused neonatal MDR study.
Semaria Solomon	2021	Prevalence and risk factors for antimicrobial resistance among newborns with gram-negative sepsis	Ethiopia	LMIC	Prospective cohort with secondary AMR analysis	Tertiary hospital (St. Paul's Hospital Millennium Medical College), inborn/outborn newborn follow-up to 60 days	Mothers and newborns enrolled at birth and followed through 60 days; analysis restricted to newborns with blood-culture-confirmed Gram-negative sepsis	Estimate prevalence of antibiotic resistance among Gram-negative neonatal sepsis isolates and identify neonatal, maternal and environmental risk factors for resistance	Not overall sepsis incidence; 119 culture-confirmed Gram-negative sepsis cases were analyzed within a larger cohort	MDR prevalence 88% (105/119); Ampicillin+gentamicin resistance 85%	This study shows that Gram-negative neonatal sepsis in Ethiopia is overwhelmingly resistant to standard first-line therapy. K. pneumoniae dominated the cohort, MDR prevalence reached 88%, and low birth weight, prematurity and especially LOS increased resistance risk. Whole-genome-informed characterization strengthened the microbiology, but effective treatment options remained limited, with amikacin and piperacillin-tazobactam appearing

											more promising than ampicillin/gentamicin. The paper is especially valuable for linking resistance patterns to clinical risk factors and to mortality from ineffective empiric therapy.
Stefani Miranda	2024	Risk factors of multidrug-resistant organisms neonatal sepsis in Surabaya tertiary referral hospital: a single-center study	Indonesia	LMIC	Cross-sectional study	Single tertiary-referral NICU (dr. Ramelan Navy Central Hospital, Surabaya)	Neonates admitted to NICU from 2020-2022 with blood culture positive for bacteria and physician-diagnosed sepsis	Identify risk factors associated with MDRO neonatal sepsis in a tertiary NICU	Among 266 blood culture requests, 113 neonates met culture-positive bacterial sepsis criteria	MDRO prevalence 80.5% (91/113)	This Indonesian NICU study found that MDROs accounted for more than four-fifths of culture-positive neonatal sepsis cases. Risk clustered around both perinatal factors (PROM, meconium-stained fluid, low birth weight, low Apgar score) and healthcare exposures (ventilation and

											prolonged hospitalization). Although the paper provides limited species-level detail in the main text, it is useful for showing how strongly NICU-related care intensity tracks with MDR sepsis risk. Its main contribution to the review is risk stratification and the need for AST-guided empirical treatment and better infection control.
Hu	2021	Klebsiella pneumoniae: Prevalence, Reservoirs, Antimicrobial Resistance, Pathogenicity, and Infection:	Global review (human, animal, food, and environmental data from multiple countries)	mixed	review	multisector review; community + hospital + food/environment reservoirs	Not neonatal cohort-specific; discusses K. pneumoniae in humans including neonates plus animal/environmental reservoirs	Summarize prevalence, reservoirs, AMR, pathogenicity, and detection of K. pneumoniae, including relevance to human infection.	NR for neonatal sepsis incidence	Describes increasing global MDR K. pneumoniae across clinical and non-clinical reservoirs	This review frames K. pneumoniae as a globally distributed MDR pathogen relevant to neonates but embedded in a broader One Health ecology. It highlights

		A Hitherto Unrecognized Zoonotic Bacterium									plasmid-mediated resistance, genomic plasticity, and possible food/animal/environment reservoirs beyond the NICU. For neonatal MDR infection research, it is most useful for understanding how Klebsiella can circulate across sectors and why hospital-only explanations may be incomplete. It also points to WGS and rapid spectroscopy as important tools for future surveillance.
Mkony	2014	Management of neonatal sepsis at Muhimbili National Hospital in Dar es Salaam:	Tanzania	LMIC	cross-sectional	hospital-based; single tertiary hospital	Neonates suspected of sepsis at Muhimbili National Hospital, July 2012-March 2013	Assess diagnostic accuracy of CRP and Rubarth newborn scale of sepsis and describe	19.2% culture-positive among suspected cases	Very high resistance to first-line antibiotics; no single summary MDR percentage reported	Among neonates with suspected sepsis in a Tanzanian tertiary hospital, one in five blood cultures was

		diagnostic accuracy of C-reactive protein and newborn scale of sepsis and antimicrobial resistance pattern of etiological bacteria						antimicrobial susceptibility of etiological bacteria.			positive and Klebsiella spp. was the leading isolate. Resistance to ampicillin and cloxacillin was essentially universal and gentamicin resistance was also substantial, suggesting standard empiric regimens may fail. The paper is especially valuable for showing how diagnostic uncertainty and delayed culture results intersect with MDR epidemiology in LMIC neonatal care. It does not provide genomic or NICU transmission data, but it clearly documents a clinically important resistance burden.

Galiza	2025	Maternal vaccination to prevent neonatal infections and combat antimicrobial resistance	Global review; authors based in UK/Uganda	mixed	review	perinatal/neonatal prevention review	Pregnant women, mothers, neonates; focuses on vaccine-preventable maternal/neonatal infections and AMR	Review how maternal immunization can prevent neonatal infections and reduce antimicrobial use/resistance.	No primary sepsis incidence estimate; cites major global burden	Describes rising MDR neonatal sepsis, especially K. pneumoniae; cites modelling of meropenem-resistant K. pneumoniae deaths	This review shifts the MDR neonatal infection conversation upstream by arguing that prevention in pregnancy can reduce neonatal antibiotic exposure and therefore AMR selection pressure. It is especially relevant because it highlights Klebsiella and E. coli maternal vaccine pipelines, NICU MDR outbreak contexts, and the high-burden regions where impact could be greatest. The paper does not provide primary clinical microbiology data, but it offers a strong prevention framework for

											narrative reviews on MDR neonatal infections. Its main contribution is linking maternal immunization to antibiotic-sparing and AMR control.
Nanayakkara	2018	Maternal vaginal colonization with selected potential pathogens of neonatal sepsis in the era of antimicrobial resistance, a single center experience from Sri Lanka	Sri Lanka	LMIC	prospective observational	single-center; obstetric unit and linked microbiology lab	Term mothers admitted for vaginal delivery and their neonates; babies needing special care were excluded	Estimate maternal and neonatal colonization with selected potential neonatal sepsis pathogens, including resistant Enterobacteriaceae, and potential mother-to-neonate transfer.	No sepsis incidence; colonization study only	MDR Klebsiella/E. coli colonization low (0.4% pre-delivery, 0.8% post-delivery, 0% neonatal); ESBL producers 1.6%, 3.1%, and 1.2% across pre/post/neonatal periods	This Sri Lankan study is important because it separates colonization from invasive disease and shows that resistant maternal-neonatal Enterobacteriaceae transfer can occur but was uncommon in this low-exposure cohort. Klebsiella colonization increased after delivery, suggesting postpartum healthcare exposure may matter even when community

											MDR carriage is low. The common ESBL determinant was blaCTX-M, and strain typing suggested occasional direct mother-to-baby transfer. For narrative reviews, the paper is especially useful for discussing maternal reservoirs, EOS biology, and the distinction between colonization and infection.
Dedeić-Ljubović	2012	Occurrence of colonization and infection with multidrug-resistant organisms in a neonatal intensive care unit	Bosnia and Herzegovina	LMIC / upper-middle (non-HIC)	surveillance / prospective observational	single NICU; hospital-based	Premature, low-birth-weight infants admitted to NICU over 6 months; surveillance nose/throat/stool cultures and clinical cultures	Determine occurrence of colonization and subsequent infection with MDROs and assess the utility of surveillance cultures.	Not general sepsis incidence; 25.5% infected with MDROs	64.3% colonized; 25.5% infected with MDROs	This NICU surveillance study is one of the most directly relevant papers in the set because it clearly distinguishes colonization from invasive MDR infection. It shows that colonized neonates were

											much more likely to become infected, with ESBL K. pneumoniae dominating fecal carriage and A. baumannii prominent in upper-airway carriage. The paper supports the idea that MDR neonatal sepsis often evolves from colonization under NICU pressure rather than appearing de novo. It also reinforces the practical value of surveillance cultures when MDROs become endemic or epidemic.
Liu	2021	Pathogens distribution and antimicrobial resistance in bloodstream infections	China	LMIC / mixed-middle income context	surveillance / retrospective descriptive	multicenter NICU; 25 tertiary hospitals	Neonates with bloodstream infection admitted to 25 NICUs, 2017-2019; EOS ≤72h, HALOS >48h after	Define pathogens and AMR profiles of EOS, hospital-acquired LOS, and community-acquired	BSI cohort only; 1092 pathogens from 1088 infants	High MDR in K. pneumoniae in HALOS (60.7%) and E. coli in EOS (44.4%)	This is one of the strongest epidemiologic papers in the set because it separates EOS, hospital-acquired LOS, and community-

		in twenty-five neonatal intensive care units in China, 2017-2019					admission, CALOS ≤48h after admission	LOS in Chinese NICUs.			acquired LOS across 25 NICUs. The data show that E. coli dominates EOS, while MDR K. pneumoniae dominates hospital-acquired LOS, with substantial cephalosporin and carbapenem resistance. For understanding MDR neonatal infection, the paper demonstrates that timing of onset is not just clinical bookkeeping- it predicts pathogen mix, resistance pattern, and mortality. It strongly supports regimen stratification and unit-specific antibiograms in NICUs.

Geleta	2024	Phenotypic bacterial epidemiology and antimicrobial resistance profiles in neonatal sepsis at Jimma medical center, Ethiopia: Insights from prospective study	Ethiopia	LMIC	cohort / prospective observational	single tertiary NICU / hospital-based	Neonates <28 days in Jimma Medical Center NICU/PNICU, systematically sampled May 2022-July 2023	Assess phenotypic bacterial epidemiology and antimicrobial resistance profiles among clinically suspected neonatal sepsis cases.	40.4% culture-proven bacterial infection among suspected sepsis	88.4% of isolates were MDR; 68.3% of Gram-negatives were ESBL producers	This Ethiopian prospective study documents an alarming clinical picture: high culture positivity, very high MDR prevalence, widespread ESBL production, and substantial mortality. Klebsiella pneumoniae dominated the microbiology and showed near-complete resistance to several cephalosporins, while ESBL-associated deaths were common. For a narrative review, the study is especially powerful as contemporary LMIC evidence that MDR neonatal sepsis is not just prevalent but clinically

											devastating. It also underscores how phenotypic surveillance alone is no longer enough and needs molecular follow-up.
Darlow	2021	Potential Antibiotics for the Treatment of Neonatal Sepsis Caused by Multidrug-Resistant Bacteria	Global review with focus on LMIC applicability	LMIC-focused review	review	therapeutics/pharmacology review	Neonates with sepsis caused by MDR bacteria; pharmacology literature on candidate antibiotics	Identify older off-patent antibiotics that could be repurposed as empiric regimens for MDR neonatal sepsis in LMICs.	Reviews global burden; 430,000-680,000 deaths annually cited	Describes high resistance to current WHO first-line regimens, especially among Gram-negatives and MRSA	This review is the strongest treatment-focused paper in the set and directly addresses what to do when standard neonatal sepsis regimens fail in MDR settings. It argues that five older, off-patent agents-amikacin, tobramycin, fosfomycin, flomoxef, and cefepime-deserve consideration for new empiric combinations, especially in LMICs. Its value lies in connecting

											resistance mechanisms to practical neonatal PK/PD and toxicity constraints. For a narrative review, it provides the therapeutic counterpart to epidemiology and transmission studies.
Flokas	2017	Prevalence of ESBL-Producing Enterobacteriaceae in Pediatric Bloodstream Infections: A Systematic Review and Meta-Analysis	Multiple countries across Africa, South America, Asia, Europe, Oceania, and multinational studies	mixed	systematic review and meta-analysis	mixed hospital settings; includes NICUs, PICUs, tertiary hospitals, multicenter cohorts	Patients <19 years; neonatal subgroup analyzed separately	Estimate prevalence, geographical distribution, and mortality associated with ESBL-producing Enterobacteriaceae BSIs in pediatric patients.	No overall neonatal sepsis incidence; focuses on ESBL-PE proportion among confirmed BSIs	ESBL-PE prevalence 9% overall; 11% among neonates	This meta-analysis quantifies how serious ESBL bloodstream infection is for neonates: prevalence is higher in neonates than older children, and mortality is roughly doubled. Its main contribution is geographic perspective, showing the burden is especially high in Africa, South America, and India. Because outbreak

											studies were excluded, the paper is useful for framing ESBL-PE as an endemic-not just epidemic-problem. It also provides strong support for stewardship and infection-control measures in neonatal units.
Johnson	2021	High Burden of Bloodstream Infections Associated With Antimicrobial Resistance and Mortality in the Neonatal Intensive Care Unit in Pune, India	India	LMIC	Prospective cohort	Multicenter tertiary NICU; hospital-based	NICU admissions (inborn and outborn) in 3 tertiary hospitals in Pune, India; followed until discharge, transfer, or death	To define the epidemiology, pathogen distribution, antimicrobial resistance, mortality burden, and risk factors of culture-proven neonatal bloodstream infection in tertiary NICUs in Pune.	EOS incidence 1.6% (55/3,341); LOS incidence 5.5% (176/3,178); LOS density 6.6 per 1,000 patient-days	Among gram-negative isolates, 45% were carbapenem-resistant; high resistance to third-generation cephalosporins and carbapenems was reported	This study shows that late-onset bloodstream infection was more common than early-onset disease in tertiary NICUs in Pune and that gram-negative organisms, especially Klebsiella-centered infections, predominated. Carbapenem resistance was frequent among gram-negative isolates and culture-proven infection was

											associated with major excess mortality. The findings strongly suggest an important role for hospital-associated transmission and device/antibiotic exposure in MDR neonatal sepsis. The paper underscores the need for local antibiograms, stewardship, and stronger infection prevention in LMIC NICUs.
Zahoor	2023	High prevalence of Panton-Valentine Leucocidin (PVL) toxin carrying MRSA and multidrug resistant gram negative bacteria in late onset neonatal	Pakistan	LMIC	Observational laboratory-based cross-sectional study / isolate surveillance	Single tertiary-care NICU; hospital-based	Neonates with suspected sepsis in a tertiary-care hospital in Peshawar, Pakistan; congenital disease cases excluded	To identify bacterial pathogens in neonatal sepsis and characterize antimicrobial resistance and virulence genes, including PVL and mecA.	No hospital incidence estimate; background cites Pakistan neonatal sepsis burden of approximately 1-4 per 1,000 live births	MDR was reported in 72.2% of S. aureus, 21% of C. freundii, 50% of P. aeruginosa, and 24% of E. coli isolates	This study highlights a late-onset neonatal sepsis pattern in a Pakistani tertiary hospital dominated by PVL-positive MRSA and multiple MDR gram-negative pathogens. The coexistence of mecA-positive

		sepsis indicate nosocomial spread in a Pakistani tertiary care hospital									MRSA, GIM-positive E. coli, and other virulence genes suggests that resistance and pathogenicity determinants are circulating together. The overall pattern strongly points to nosocomial spread within the NICU rather than isolated sporadic cases. The paper emphasizes the need for stronger molecular surveillance and infection control in high-burden South Asian settings.
Lona-Reyes	2025	Microorganisms identified in neonatal sepsis and their antimicrobial resistance in a	Mexico	LMIC	Cross-sectional study nested in a cohort	Single-center NICU; hospital-based	Newborns born and hospitalized at NHCGJIM in Jalisco, Mexico, from October 2021 to October 2023	To describe the microorganisms causing neonatal sepsis and their antimicrobial resistance profiles in a hospital in	EOS incidence 7.1 per 1,000 live births; LOS incidence 25.5 per 1,000 live births	No single MDR prevalence reported, but high resistance among Enterobacterales and high oxacillin resistance among staphylococci were documented	This Mexican NICU study shows a high burden of late-onset neonatal sepsis, with Enterobacterales-especially Klebsiella pneumoniae-dominating

		hospital in Western Mexico						western Mexico.			the microbiological landscape. Resistance to standard first-line EOS therapy was substantial, and oxacillin resistance among staphylococci was particularly high in LOS. The study is especially valuable because it demonstrates how local NICU antibiograms can directly change empirical treatment policies. It also highlights the importance of ongoing center-specific surveillance in neonatal AMR.
Thatrimontrichai	2019	Multidrug resistant Gram negative bacilli sepsis from a	Thailand	LMIC	Retrospective case-case-control study	Single tertiary referral NICU; hospital-based	Neonates with blood or CSF culture-proven gram-negative	To identify risk factors and outcomes of multidrug-resistant gram-	No birth-cohort incidence reported; among gram-negative	MDRGNB represented 64.2% of gram-negative sepsis episodes	This study presents MDR gram-negative neonatal sepsis as a long-standing NICU

		neonatal intensive care unit					sepsis in a tertiary referral NICU in southern Thailand, 1991-2016	negative bacilli sepsis in a NICU.	sepsis episodes, MDRGNB accounted for 64.2%		ecological problem rather than a series of isolated resistant episodes. Prematurity, referral from outside hospitals, invasive device exposure, and prior antibiotic use were the major risk signals for MDRGNB sepsis. Clinically, MDRGNB infection carried a heavy burden of death, septic shock, neurologic sequelae, prolonged hospitalization, and higher costs. The paper strongly supports stewardship and prevention of hospital transmission as central priorities.

Dharmapalan	2017	High Reported Rates of Antimicrobial Resistance in Indian Neonatal and Pediatric Blood Stream Infections	India	LMIC	Literature review	Predominantly tertiary hospital and NICU studies from India	Hospitalized children from birth to 18 years in India; many included studies focused on NICUs and neonatal bloodstream infections	To summarize antimicrobial resistance patterns in Indian neonatal and pediatric bloodstream isolates.	Across included studies, 29.1% of blood cultures were positive; the review did not calculate a pooled neonatal sepsis incidence	Very high resistance across common neonatal pathogens; MRSA around 50% overall and major resistance to ampicillin, aminoglycosides, and cephalosporins among gram-negative organisms	This review shows that resistance to standard neonatal first-line therapy is alarmingly high across Indian pediatric and neonatal bloodstream isolates, especially among Klebsiella and E. coli. Although the source literature is heterogeneous, the overall signal is consistent: Indian tertiary NICUs face a major MRSA- and gram-negative-dominated AMR problem. The paper is especially useful because it links microbiology to system-level drivers such as weak surveillance, poor infection control, and

											unregulated antibiotic use. It supports the need for standardized national neonatal AMR surveillance and better treatment guidance.
Berberian	2019	Multidrug resistant Gram-negative infections in neonatology	Argentina	LMIC	Retrospective observational cohort	Single tertiary pediatric hospital NICU; hospital-based referral unit	Median gestational age 35 weeks; median birth weight 2070 g; 57% preterm; all had underlying disease; many surgical referrals	To describe the epidemiological, clinical, and microbiological characteristics of carbapenem-resistant MDR Gram-negative infections in a tertiary NICU and identify factors related to infection and mortality.	NR; study focused on 21 carbapenem-resistant MDR Gram-negative infections rather than unit-wide sepsis incidence.	100% of included cases were carbapenem-resistant MDR Gram-negative infections.	In this tertiary Argentine NICU, carbapenem-resistant MDR Gram-negative infection was largely a late, device-associated problem dominated by A. baumannii. Prior broad-spectrum antibiotic exposure, invasive support, central lines, and prolonged hospitalization were common. Intestinal colonization with the same organism was documented in some

											infants, suggesting colonization pressure and hospital transmission. The study mainly supports strict infection control and careful antimicrobial stewardship in high-risk neonatal units.
Wei	2015	Multidrug-resistant Acinetobacter baumannii infection among neonates in a neonatal intensive care unit at a medical center in central Taiwan	Taiwan	HIC	Retrospective case series with outbreak investigation elements	Single tertiary NICU, hospital-based	Neonates with at least one MDRAB infection episode admitted between 2010 and 2013 in a 20-bed NICU.	To evaluate mortality risk factors in neonates with MDRAB infection and describe outbreak/transmission patterns in the NICU.	NR for overall neonatal sepsis incidence; study reported 59 neonates with MDRAB infection over 4 years.	100% of included isolates/cases were multidrug-resistant A. baumannii.	This Taiwan NICU study shows that MDR Acinetobacter baumannii can behave as an endemic-outbreak pathogen with clear environmental persistence and clonal spread. Bloodstream infection was common and mortality was substantial, especially when active therapy was delayed and when severe cytopenias were present.

											PFGE linked clinical and environmental isolates, underscoring the role of horizontal transmission in the NICU. The study supports aggressive infection-control action and timely use of active agents such as colistin.
Awad	2016	Multidrug-resistant organisms in neonatal sepsis in two tertiary neonatal ICUs, Egypt	Egypt	LMIC	Retrospective surveillance / observational study	Two tertiary university NICUs; hospital-based	Culture-proven neonatal sepsis from two Cairo tertiary NICUs; mean birth weight 2.67 kg; 43.8% preterm; 35.4% EOS and 64.6% LOS.	To define the bacteriological profile, antimicrobial resistance patterns, risk factors, and outcomes of neonatal sepsis in two tertiary Egyptian NICUs.	33.3% of admissions were clinically diagnosed with sepsis; 32.25% of suspected sepsis cases were culture proven; 240 culture-proven cases among 2237 admissions.	77% overall MDR; 83.4% of Gram-negative isolates and 60% of Gram-positive isolates were MDR.	This Egyptian study documents an extremely high MDR burden in neonatal sepsis, with Gram-negative organisms dominating both early- and late-onset disease. E. coli was the leading pathogen, and most deaths occurred in infections caused by MDR organisms. Maternal risk

											factors shaped EOS, whereas invasive care and healthcare exposure shaped LOS. The study strongly argues that empirical regimens must be revised using local antibiograms and ongoing surveillance.
Jain	2025	Multidrug-resistant sepsis in special newborn care units in five district hospitals in India: a prospective cohort study	India	LMIC	Prospective cohort study	Multicenter special newborn care units (district hospitals); hospital-based	3972 inborn and 2640 outborn neonates admitted to five district hospital special newborn care units; mean gestation 37.1 weeks; mean birth weight 2540 g.	To estimate the incidence of culture-positive neonatal sepsis, describe the pathogen and resistance profile, and assess variation across district-level newborn units.	Overall incidence of culture-positive sepsis 3.2% (site range 0.6-10.0%); 50.8% met clinical sepsis criteria.	Approximately 75-88% MDR among major Gram-negative pathogens depending on species.	This multicenter Indian cohort shows that multidrug-resistant neonatal sepsis is a major district-hospital problem, not only a tertiary NICU problem. Gram-negative pathogens such as K. pneumoniae and E. coli dominated, and resistance rates were very high. Outborn babies,

											overcrowded units, poor staffing, and limited microbiology capacity appeared to sustain the burden. The study's strongest message is that district-level diagnostics, stewardship, and infection prevention must be strengthened.
Basu	2015	Multidrug-resistant Trichosporon: an unusual fungal sepsis in preterm neonates	India	LMIC	Case series / outbreak cluster report	Single NICU; hospital-based	Three extremely low birth weight preterm neonates (27-28 weeks; 900-980 g) with late-onset sepsis over one week.	To report a fatal cluster of multidrug-resistant Trichosporon asahii late-onset sepsis in preterm neonates and emphasize its unusual resistance profile.	No study denominator incidence provided; article cites external literature on Trichosporon fungemia.	All 3 isolates showed resistance to amphotericin B and fluconazole and susceptibility to voriconazole.	This case series expands the MDR neonatal infection discussion beyond bacteria by showing that resistant fungal late-onset sepsis can be devastating in preterm ELBW infants. All three neonates died, and the decisive problem was that the organism was

											resistant to amphotericin B and fluconazole while voriconazole was not immediately available. The clinical presentation initially resembled routine NICU sepsis, which can delay effective treatment. The report highlights the need for high suspicion for unusual fungal pathogens and rapid access to active antifungal therapy.
Ambreen	2020	Efficacy of colistin in multidrug-resistant neonatal sepsis: experience from a tertiary care center in Karachi, Pakistan	Pakistan	LMIC	Retrospective record review / observational cohort	Single tertiary-care NICU, Aga Khan University Hospital, Karachi	Mostly male, preterm and low-birth-weight neonates with severe sepsis/septic shock receiving IV, inhaled and/or intrathecal colistin	To evaluate efficacy, predictors of response, microbial clearance, survival, and safety of colistin in neonatal MDR-GNB sepsis	Not a population-incidence study; within the treated cohort 120/153 (78.4%) had culture-	93/120 (77.5%) culture-proven cases had MDR-GNB; 84 were colistin-sensitive	This Pakistani tertiary-NICU study shows how frequently neonatal MDR Gram-negative sepsis requires salvage therapy with colistin. Survival was

									proven sepsis		better when colistin was started early and when pathogens were colistin-susceptible, while K. pneumoniae and A. baumannii predominated. Toxicity was present but not overwhelming, supporting cautious use where alternatives are limited. The study underscores the treatment challenges faced in LMIC NICUs with high MDR burdens.
Uzunovic	2014	Emergency (clonal spread) of methicillin-resistant Staphylococcus aureus (MRSA), extended spectrum (ESBL)- and AmpC	Bosnia and Herzegovina	LMIC	Hospital-based surveillance / molecular epidemiology / outbreak investigation	Pediatric department and affiliated laboratories; hospital-based, not NICU-specific	Children with MRSA or beta-lactamase-producing Gram-negative infections; substantial newborn representation	To determine prevalence and molecular characteristics of MRSA, ESBL and plasmid-mediated AmpC Gram-negative	Not a neonatal-sepsis incidence study	Among targeted pediatric isolates, MRSA and ESBL/AmpC producers were common; ESBL prevalence among children reported as 7.8%	This Bosnian pediatric study is important because many resistant isolates came from newborns and clear clonal spread was demonstrated. MRSA CC152 (Balkan clone)

		beta-lactamase-producing Gram-negative bacteria infections at Pediatric Department, Bosnia and Herzegovina						bacteria in children			and CTX-M-15-producing K. pneumoniae were the dominant resistant lineages. The paper shows that neonatal and pediatric MDR problems are not only about individual cases but also about transmission networks. It strongly supports molecular surveillance plus infection-control measures to prevent outbreaks.
Hashmi	2020	Emerging Antimicrobial Resistance in Neonatal Sepsis	Pakistan	LMIC	Descriptive retrospective study	Single NICU, Fauji Foundation Hospital, Rawalpindi/Islamabad	NICU neonates admitted with suspected sepsis over May 2017-April 2019; mostly newborns, male:female 1.36:1	To determine the frequency and antimicrobial sensitivity pattern of organisms causing neonatal sepsis	Blood-culture positivity 7.4% among 1,070 neonates with sepsis	No formal MDR proportion given; many Klebsiella and Acinetobacter isolates were highly resistant and often susceptible only to colomycin	This Pakistani NICU study shows that culture-positive neonatal sepsis was uncommon but highly resistant when present. Gram-negative organisms, especially

											Acinetobacter and Klebsiella, slightly outnumbered Gram-positives, and several resistant strains were reportedly susceptible only to colomycin. The paper highlights how low culture yield and rapidly changing resistance patterns complicate empirical therapy. It supports routine local antibiogram updates for neonatal units.
Demirbuga	2024	Emerging importance of multidrug-resistant Stenotrophomonas maltophilia infections in neonatal intensive care unit	Turkey	LMIC	Retrospective observational study	Single tertiary-center NICU, Istanbul University	Hospitalized neonates in NICU (2020-2021) with S. maltophilia isolated from lower respiratory tract, urine or peritoneal fluid	To assess incidence, clinical features, susceptibility pattern and treatment of S. maltophilia healthcare-associated infection/col	Not a general sepsis-incidence study; S. maltophilia represented 24% of hospital-acquired infectious agents in the unit	All cases involved MDR S. maltophilia; 40% were colonization and 60% fulfilled infection criteria	This Turkish NICU study shows that S. maltophilia is emerging as a clinically relevant MDR pathogen, especially in heavily instrumented neonates. The paper clearly separates

		in a tertiary center in Turkey						onization in the NICU			colonization from infection, with ventilator-associated pneumonia dominating and no bacteremia detected. Rising TMP-SMX resistance and limited neonatal dosing options make management difficult. It reminds reviewers that not all NICU MDR threats are the classic Klebsiella/Acinetobacter bloodstream pathogens.
Zou	2021	Emerging Threat of Multidrug Resistant Pathogens From Neonatal Sepsis	China	LMIC	Multicenter retrospective surveillance /cohort with nested case-control analyses	Four Women and Children's hospitals / neonatal wards in Southwest China	Hospitalized neonates <=28 days with culture-proven sepsis/bacteremia from four hospitals; mean gestation 35.68 weeks, mean birth weight 2517.8 g	To characterize pathogens, resistance phenotypes/genes, sequence types, and risk factors associated with MDR neonatal sepsis in Southwest China	Hospital-based culture-proven cohort; no admission-based population incidence reported	EOS pathogens: 64.86% MDR; LOS pathogens: 78.33% MDR	This multicenter Chinese study is one of the most informative molecular papers in the set. It shows that E. coli dominated EOS, K. pneumoniae dominated LOS, and

											MDR was very common in both groups, especially LOS. High-risk clones (E. coli ST167 and K. pneumoniae ST11) plus blaCTX-M and NDM-1 help explain the resistance burden. The study links prior antibiotic exposure and LOS to MDR infection, making it highly relevant for both epidemiology and stewardship sections of a review.
Kumar	2025	Emerging trend of multidrug-resistant organisms causing sepsis in ventilated newborns	India	LMIC	Observational cross-sectional study	Single neonatal ICU, MGM Medical College/LSK Hospital, Bihar	Mechanically ventilated neonates with sepsis-positive screens admitted to NICU; congenital anomalies and very/extreme	To identify organisms causing sepsis in ventilated newborns, determine prevalence of MDR isolates, and describe antibiotic	47.02% culture positivity among ventilated newborns studied	53/79 (67.08%) isolates were MDR	This Indian NICU study concentrates on one of the highest-risk groups: ventilated newborns. It found very high culture positivity and a striking

							ly low birth weight were excluded	resistance patterns			MDR rate, with Gram-negative organisms, especially Pseudomonas, slightly predominating and many EONS isolates already resistant. The study is useful for showing how critical-care exposures can coincide with a narrow therapeutic window. It supports including ventilated neonates as a distinct high-risk subgroup in narrative reviews on MDR sepsis.
Odoyo	2023	Environmental contamination across multiple hospital departments with multidrug-resistant bacteria pose an elevated risk of	Kenya	LMIC	Multicenter cross-sectional environmental surveillance study	Five hospitals; high-touch surfaces sampled across newborn, maternity, pediatric, surgical, general and outpatient departments	Environmental rather than patient-level study; focused on hospital surface contamination including newborn units	To determine the burden and types of MDR ESKAPEE organisms contaminating high-touch hospital surfaces in Kenyan hospitals	Not a patient-level neonatal sepsis study	78/617 (12.6%) surfaces contaminated with MDR ESKAPEE	Although not a direct neonatal sepsis cohort, this Kenyan study is highly relevant for NICU transmission biology. It shows that newborn incubators,

		healthcare-associated infections in Kenyan hospitals									baby cots, beddings and sinks can act as reservoirs for MDR ESKAPEE organisms, especially Acinetobacter and Klebsiella. The work helps explain how healthcare-associated neonatal MDR infections may be sustained even when patient-level data are incomplete. It is particularly useful for the infection-prevention section of a review.
Lama	2021	Genomic analysis of a multidrug-resistant Brucella anthropi strain isolated from a 4-day-old neonatal sepsis patient	India	LMIC	Case report / genome note	Single neonatal sepsis case; hospital-based microbiology/genomics analysis	A 4-day-old neonate with blood-culture-positive sepsis	To characterize genome features, antimicrobial resistance and pathogenic potential of an MDR B. anthropi isolate from neonatal sepsis	NA - single case report	NA - single MDR isolate case	This paper is valuable because it broadens the pathogen spectrum beyond the usual neonatal MDR organisms. Using hybrid whole-genome sequencing, it identified a

											rare MDR B. anthropi bloodstream isolate with multiple AMR, virulence and mobile-element features. The case underscores how unusual neonatal pathogens may be overlooked or misidentified without advanced diagnostics. It is especially relevant for sections on genomics and emerging opportunistic pathogens.
Beshah	2023	High burden of ESBL and carbapenemase-producing gram-negative bacteria in bloodstream infection patients at a tertiary care	Ethiopia	LMIC	Institution-based cross-sectional study	Tertiary-care hospital (Tikur Anbessa Specialized Hospital), all-age bloodstream infection patients; includes NICU/pediatric wards	All age groups with suspected bloodstream infection; neonatal and NICU subgroups were analysed as high-risk categories	To determine the magnitude of ESBL, AmpC and carbapenemase-producing Gram-negative bacteria and associated risk factors in	Not neonatal-sepsis specific	Among Gram-negative isolates: ESBL 54.0%, carbapenemase 25.7%, ESBL+AmpC 6.9%; 84.4% produced at least one drug-hydrolyzing enzyme	This Ethiopian bloodstream-infection study is not neonatal-only, but it is highly relevant because neonates and NICU contexts emerged as high-risk strata. ESBL and

		hospital in Addis Ababa, Ethiopia						bloodstream infections			carbapenemase burdens were extremely high, K. pneumoniae dominated, and Acinetobacter showed particularly alarming carbapenemase/pan-drug resistance. The paper helps frame how severe the broader hospital Gram-negative AMR ecosystem can be around neonatal units. It is especially useful for the resistance-mechanisms and health-system sections of a review.
Aradhya	2025	High prevalence of antimicrobial resistance to initial empirical antibiotic therapy in	India	LMIC	Prospective multicentre observational study	Six tertiary-care NICUs across Bengaluru, South India	Inborn and outborn neonates with positive blood cultures collected prospectively from June 2020 to May	To describe burden, organisms and resistance patterns in neonatal sepsis and assess how often initial	3.5% incidence among admissions	Overall MDR among Gram-negative organisms 26%; organism-specific MDR: Acinetobacter 81%, Klebsiella 48%, E. coli 45%	This Bengaluru network study is one of the clearest practical papers in the set because it links resistance

		neonatal sepsis in Bengaluru, India-a multicentre study					2022; mean birth weight 2165 g, 73% outborn	empirical therapy was on-target			directly to empirical treatment failure. Gram-negative LOS dominated, Klebsiella was most common, Acinetobacter was the most MDR, and initial therapy was on-target in only 48% of episodes. Mortality was significantly higher when empirical therapy missed the pathogen. The paper strongly supports local antibiograms and stewardship as central parts of neonatal MDR sepsis management.
Seni	2019	Deciphering risk factors for blood stream infections, bacteria species and antimicro	Tanzania	LMIC	Multiple cross-sectional analytical study	Multicentre, hospital-based cascade of referral care (tertiary, regional, district; urban and rural)	Children <5 years; median age 9 months; 346 neonates, 209 infants 2-12 months, 395 children 13-60 months; 4 hospitals in	To define BSI prevalence, bacterial species, AMR profiles, and risk factors for BSI and mortality across	Bloodstream infection prevalence 14.2% overall; age-specific BSI prevalenc	77.8% (105/135) of isolates were MDR; 79.0% of Enterobacteriaceae were third-generation cephalosporin resistant.	This multicentre Tanzanian study showed a high BSI burden in children under five, with neonates carrying the

		bial resistance profiles among children under five years of age in North-Western Tanzania: a multicentre study in a cascade of referral health care system					north-western Tanzania	referral-level hospitals.	e 25.4% in neonates, 5.7% in infants 2-12 months, 8.9% in children >12 months.		greatest risk and tertiary-hospital isolates showing the highest resistance burden. MDR K. pneumoniae dominated the microbiology, and ESBL phenotypes were common among Enterobacteriaceae. Mortality was 6.6% overall and was concentrated among neonates, tertiary-center patients, and culture-positive cases. The paper strongly supports routine culture/AST and targeted prevention efforts focused on neonatal referral care.
Pons	2025	Direct economic costs related to antimicro	Peru	LMIC	Cross-sectional economic analysis using	Single-center perinatal referral hospital (Instituto	Newborns <28 days with blood culture-confirmed	To estimate direct economic costs associated	No population incidence reported;	225/288 (78.1%) MDR isolates; MDR strains had higher mean direct costs than non-MDR strains.	This Peruvian study adds an important economic perspective by

		bial resistance in bloodstream infections isolated from newborns in a perinatal hospital in Peru			micro-costing bottom-up approach	Nacional Materno Perinatal, Lima)	bacteremia/neonatal sepsis, January 2017 to June 2018; 91 EOS and 197 LOS cases.	with microorganisms and antimicrobial resistance among neonatal bloodstream infections.	study included 288 culture-positive neonatal BSI cases.		showing that MDR neonatal BSIs are more expensive to treat than non-MDR infections. LOS was the strongest cost amplifier, and non-fermenting Gram-negative organisms such as Acinetobacter produced the highest per-case costs. Although clinical outcomes were not detailed, the paper demonstrates that AMR imposes a clear system-level financial burden in neonatal care. It supports stronger AMR control policies in LMIC perinatal hospitals.
Hadžić	2020	Epidemiology of neonatal	Bosnia and	LMIC	Retrospective cohort study	Single-center level III NICU, hospital-based	Consecutive neonates treated in the	To analyze the epidemiolog	Clinically confirmed sepsis	MDR pathogens caused sepsis in 22/921 admissions (2.38%) and	This Bosnian NICU study shows that

		sepsis caused by multidrug resistant pathogens in a neonatal intensive care unit level 3	Herzego vina				NICU of the Paediatric Clinic, Tuzla, during 2016-2018; MDR cases compared with non-MDR sepsis controls.	y, risk factors, clinical profile, microbiology and outcomes of MDR neonatal sepsis in a level III NICU.	42.9%; blood culture-confirme d sepsis 20.3% among NICU admissio ns.	22/187 culture-proven sepsis cases (~11.8%).	MDR neonatal sepsis, although numerically infrequent, is clinically severe. It was concentrated in premature, low-birth-weight neonates, was usually late-onset, and carried far higher mortality than non-MDR sepsis. Gram-negative organisms-especially Acinetobacter-dominated the MDR microbiology and showed substantial resistance to standard agents. The study argues for center-specific surveillance and rapid, rational antibiotic adjustment in NICU practice.
Putri	202 5	Epidemiol ogy of	Indonesi a	LMIC	Multicentre retrospectiv	Three urban tertiary referral	Hospitalized neonates ≤28	To define the epidemiolog	Significan t	Overall MDR prevalence not	This Indonesian

		sepsis in hospitalised neonates in Indonesia: high burden of multidrug-resistant infections reveals poor coverage provided by recommended antibiotic regimens			e observational microbiology study plus antibiotic point prevalence survey	hospitals (Jakarta, Bandung, Surabaya); hospital-based neonatal sepsis surveillance	days with positive blood cultures during 2019-2020; blood cultures obtained for clinical instability or transfer from peripheral facilities.	y and AMR burden of neonatal sepsis and estimate coverage of commonly recommended empiric regimens using WISCA.	pathogens were isolated from 16% (858/5439) of neonatal blood cultures analyzed.	expressed as a single percentage, but dominant Gram-negative pathogens showed very high non-susceptibility to first- and second-line WHO regimens; study concludes high MDR burden.	multicentre study demonstrates that hospitalised neonatal sepsis is overwhelmingly driven by Gram-negative pathogens, especially Klebsiella and Acinetobacter. WHO-recommended first- and second-line regimens covered only about one quarter of cases, explaining heavy reliance on Watch/Reserve antibiotics. The work is especially valuable because it translates susceptibility data into syndrome-level treatment coverage using WISCA. It strongly supports both

											new antibiotic development and better infection-control strategies in high-burden Asian referral hospitals.
Gadisa	2024	Epidemiology, antimicrobial resistance profile, associated risk factors and management of carbapenem resistant Klebsiella pneumoniae in children under 5 with suspected sepsis in Ethiopia	Ethiopia	LMIC	Prospective multicenter study	Three tertiary hospitals across central, western and southern Ethiopia; NICU/PICU, pediatric wards, emergency and other departments	Under-5 children with suspected sepsis from June 2021 to December 2023; included neonates, infants and older children; 34.2% of positive isolates were from neonates.	To determine epidemiology, AMR profile, risk factors and management of carbapenem-resistant K. pneumoniae sepsis in under-5 children.	K. pneumoniae isolated in 530/2483 (21.4%) suspected sepsis cases; neonates and infants were the most affected age groups.	Among K. pneumoniae isolates, 95.7% MDR, 25.9% XDR and 11.4% PDR; 47.4% carbapenemase-producing and 92.1% ESBL-producing.	This Ethiopian multicenter study documents an alarmingly high burden of ESBL- and carbapenemase-producing K. pneumoniae among under-5 sepsis cases, with neonates and infants especially affected. LOS, ICU exposure, invasive procedures, prematurity, low birth weight and multiple maternal/perinatal factors all contributed to risk. Standard WHO regimens appeared inadequate against this

											resistance profile, and most culture-positive patients required therapy changes after AST. The paper is highly relevant for reviews of MDR neonatal infection because it links microbiology, clinical severity and health-system vulnerability in a low-resource setting.
Lona Reyes	2015	Etiology and antimicrobial resistance patterns in early and late neonatal sepsis in a Neonatal Intensive Care Unit	Mexico	LMIC	other (cross-sectional)	NICU; single-center tertiary hospital; hospital-based	Hospitalized neonates with culture-proven blood or CSF infection. EONS <72 h; nosocomial LONS ≥72 h to 90 days. Outborn/rehospitalized infants excluded.	Describe etiologic agents and antimicrobial susceptibility in EONS and LONS at a NICU.	EONS incidence 4.7 events/1,000 newborns; confirmed nosocomial LONS in 10.7% of hospitalized infants.	ESBL among Enterobacteriaceae: 6.5% in EONS vs 40.0% in LONS; oxacillin resistance in Staphylococcus spp.: 65.5%.	This Mexican NICU study showed a clear EOS/LOS split, with Enterobacteriaceae dominating both syndromes but Staphylococcus spp. and yeasts concentrated in nosocomial LONS. K. pneumoniae

											was the most common species, and ESBL Enterobacteriaceae were far more common in LONS (40.0%) than EONS (6.5%). Oxacillin resistance among Staphylococcus spp. was high (65.5%), whereas amikacin, piperacillin-tazobactam, and meropenem retained activity against most Enterobacteriaceae. The paper supports NICU-specific antimicrobial surveillance to guide empiric therapy in resource-constrained settings.
Gusland	2025	Etiology and antimicrobial resistance patterns of	Ethiopia	LMIC	cohort (prospective observational	Single-center tertiary hospital; NICU and pediatric ward; hospital-based	Infants 0-59 days old admitted with sepsis/PSBI at Jimma	Define etiology, susceptibility, and outcomes of PSBI/sepsis	Culture positivity was 76.0% among infants	Very high resistance to first- and second-line agents; Klebsiella spp. showed resistance to at least one commonly used antibiotic in 87%	In Jimma, microbiologically confirmed PSBI was dominated by Klebsiella,

		sepsis in infants 0-59 days old in Jimma, Ethiopia: a longitudinal study			longitudinal)		University Hospital. Presumed hospital-acquired infections were excluded. Most infants were >72 h old at admission (93.6%).	in Jimma and assess whether WHO guideline-based empiric therapy matched local susceptibility patterns.	who had blood cultures; population incidence was not estimated.	and to first-line empiric agents in 82%.	CoNS, and S. aureus, with very high resistance to first- and second-line WHO-recommended antibiotics. Empiric ampicillin/gentamicin appeared inadequate for many infants, and nearly half of cultured cases may have received inappropriate initial therapy. Mortality was substantial, especially in Klebsiella-associated infection, while short-term outcomes among survivors were generally good at 30 days. The study strongly supports local antibiograms, upgraded microbiology capacity, and

											Ethiopia-specific empiric treatment protocols.
Yu	2021	Etiology, antimicrobial resistance, and risk factors of neonatal sepsis in China: a systematic review and meta-analysis from data of 30 years	China	mixed	review (systematic review and meta-analysis)	Multicenter hospital-based studies from tertiary referral facilities/large national hospitals	Culture-proven neonatal sepsis/meningitis from blood or CSF across multiple Chinese regions; included EOS and LOS subgroups when reported.	Evaluate regional etiology, AMR patterns, and risk factors of neonatal sepsis in China.	Intro cites 2,202 neonatal sepsis cases per 100,000 live births and blood-culture positivity of 17.0% among suspected neonatal sepsis in mainland China.	No single pooled MDR prevalence was given, but the review emphasizes increasing multidrug resistance; high pooled resistance was seen in CoNS and Klebsiella, and carbapenem resistance was reported in several studies.	Across 29 Chinese studies, CoNS, E. coli, and Klebsiella spp. dominated neonatal sepsis, with a clear EOS/LOS split: GBS led EOS while CoNS and Klebsiella were more prominent in LOS. Resistance was especially high in CoNS and Klebsiella, including near-universal resistance of Klebsiella to ampicillin and widespread reported carbapenem resistance in some studies. The strongest associated risk factors were invasive NICU care and antibiotic exposure, particularly

											total parenteral nutrition, PICC use, and multiple antibiotics. Overall, the review shows that MDR neonatal sepsis in China is heterogeneous by region and closely linked to NICU practices.
Darlow	2022	Flomoxef and fosfomycin in combination for the treatment of neonatal sepsis in the setting of highly prevalent antimicrobial resistance	United Kingdom / Switzerland / United States research institutions; laboratory isolates from multiple reference collections	mixed	other (preclinical in vitro / hollow-fibre infection model study)	Laboratory-based experimental pharmacodynamic study	No infant cohort. Study used bacterial isolates representing neonatal sepsis pathogens and resistance mechanisms relevant to AMR-prevalent settings.	Assess whether flomoxef plus fosfomycin could serve as an alternative empirical regimen for neonatal sepsis in settings with high AMR.	No study-derived incidence. Background notes neonatal sepsis causes an estimated 430,000-680,000 deaths annually.	No cohort prevalence calculated. Background cites approximately 80% resistance to amoxicillin and 60% to gentamicin in LMIC neonatal sepsis studies.	This preclinical study does not describe infant outcomes, but it is highly relevant mechanistically for MDR neonatal sepsis. In AMR-representative laboratory models, flomoxef plus fosfomycin showed synergistic killing and reduced emergence of fosfomycin resistance, especially

											against resistant Enterobacterales for which monotherapy often failed. Because both drugs are off-patent and licensed for neonatal use, the combination could be a practical empiric option in LMIC settings with high ampicillin/gentamicin resistance. Clinical validation is still required, particularly for real-world neonatal sepsis and meningitis.
Investigators of the Delhi Neonatal Infection Study (DeNIS) collaboration	2016	Characterisation and antimicrobial resistance of sepsis pathogens in neonates born in tertiary care centres in	India (Delhi)	LMIC	Prospective multicentre cohort	Multicenter tertiary hospital-based NICUs	Inborn neonates delivered in three tertiary care centres in Delhi and admitted to NICU for any indication; mean birthweight 2211 g; 44.2%	To describe incidence, organism profile, and antimicrobial resistance patterns of neonatal sepsis in tertiary hospitals in Delhi.	Total sepsis 14.3% of NICU admissions (21.8/1000 live births); culture-positive sepsis 6.2% of	High species-specific MDR rates among Gram-negatives: Acinetobacter spp 82%, Klebsiella spp 54%, E. coli 38%; methicillin resistance in CONS 61% and S. aureus 38%.	This large multicentre Delhi cohort shows that neonatal sepsis in tertiary NICUs is both common and highly lethal, with most episodes occurring in

		Delhi, India: a cohort study					preterm; 59.9% low birthweight.		NICU admissions (9.5/1000 live births).		the first 72 hours of life. The microbiology is dominated by Gram-negative, hospital-associated organisms- especially Acinetobacter and Klebsiella- with striking multidrug resistance, including resistance to reserve antibiotics. EOS and LOS had very similar pathogen profiles, suggesting early acquisition of nosocomial organisms rather than classic maternal flora. The findings argue for stronger infection prevention, surveillance, and focused research into the origins of

											early neonatal MDR sepsis.
Bandyopadhyay	2018	Distribution, antimicrobial resistance and predictors of mortality in neonatal sepsis	India (New Delhi)	LMIC	Retrospective cohort	Single-center level III tertiary hospital NICU	NICU-admitted neonates with culture-proven sepsis at Kalawati Saran Children's Hospital/Lady Hardinge Medical College, Feb 2012-Feb 2014; median birthweight 1340 g; median gestation 33 weeks; 77% preterm; 76% low birthweight.	To investigate etiologic agents, antimicrobial resistance patterns, and predictors of mortality in culture-proven neonatal sepsis.	No population incidence denominator reported within cohort; 183 culture-positive sepsis cases identified. Intro cites hospital-based Indian incidence ~30/1000 live births.	High species-specific MDR among Gram-negatives: Klebsiella 49.1%, E. coli 50%, Citrobacter 50%, Acinetobacter 28.5%, Pseudomonas 100%; methicillin resistance in CONS 16.6%, S. aureus 24.4%, Enterococcus spp 62.5%; VRE in 25% of Enterococcus isolates.	This single-center Indian NICU cohort found that culture-proven neonatal sepsis remained highly lethal, especially among preterm and low-birthweight infants. Klebsiella and staphylococci dominated the microbiology, and resistance extended even to reserve antibiotics, with nearly half of Klebsiella isolates classified as MDR. Early- and late-onset sepsis had similar pathogen profiles, reinforcing concern for healthcare-associated acquisition very early in

											life. Invasive ventilation worsened mortality risk, whereas breast milk feeding appeared protective.
Darlow	2022	Flomoxef for neonates: extending options for treatment of neonatal sepsis caused by ESBL-producing Enterobacterales	Systematic review of published neonatal flomoxef studies, largely from East Asia (Japan and surrounding licensed-use settings); modelling performed in the UK	Mixed	Systematic review with population pharmacokinetic modelling and Monte Carlo simulation	Hospital-based neonatal studies aggregated from multiple published cohorts; PK modelling study	Infants <6 months from published studies receiving flomoxef for suspected/confirmed infection or prophylaxis; major indications included pneumonia, chorioamnionitis, neonatal sepsis, SSTI and UTI.	To summarize published neonatal flomoxef clinical data, build the first neonatal population PK model, and assess age-appropriate regimens for ESBL-prevalent neonatal sepsis settings.	No study-specific incidence reported; background notes global neonatal sepsis causes an estimated 430,000-680,000 deaths annually, mostly in LMICs.	Not estimated within included neonatal studies; article focuses on ESBL-prevalent settings and rising AMR compromising WHO first-line therapy.	This paper does not describe epidemiology within a single NICU but instead evaluates a potential therapeutic response to MDR neonatal sepsis, especially ESBL-producing Enterobacterales. By synthesizing historical neonatal data and building the first flomoxef PopPK model, it proposes age-stratified regimens that may achieve useful PK/PD targets with low observed toxicity. The study is

											especially relevant for LMIC settings where standard ampicillin-gentamicin therapy is increasingly undermined by ESBLs and aminoglycoside resistance. Its main value is therapeutic and pharmacologic rather than epidemiologic or molecular.
Sharma	2015	Bacteriological profile and clinical predictors of ESBL neonatal sepsis	India (Hyderabad)	LMIC	Retrospective observational cohort	Single-center level III tertiary hospital NICU	Newborns admitted to the NICU from Jan 2013 to Aug 2014 with perinatal risk factors or clinical signs of sepsis and positive blood culture; EOS defined as ≤72 h and LOS as >72 h to day 30.	To define the bacteriologic profile, incidence of ESBL infection, and clinical/perinatal risk factors for early- and late-onset ESBL Gram-negative neonatal sepsis in a cephalosporin-restricted NICU.	Culture-positive sepsis occurred in 14% of suspected sepsis episodes (209/1449) among NICU admissions; 24% of sepsis cases were EOS and 76% LOS.	Among Gram-negative sepsis, ESBL prevalence was 60.7% overall (101/167); 44.7% in early-onset Gram-negative sepsis (17/38) and 65% in late-onset Gram-negative sepsis (84/129). Among all culture-positive sepsis cases, ESBL Gram-negative sepsis accounted for ~48% (101/209).	This Hyderabad NICU study shows that ESBL Gram-negative sepsis is highly prevalent, especially in LOS, even when cephalosporin use in newborns is restricted. Klebsiella dominated ESBL infections, while E. coli had the highest

											organism-specific ESBL rate. For EOS, maternal cephalosporin exposure and preterm PROM were the clearest predictors, linking maternal/perinatal care to neonatal resistance patterns. The paper suggests that antibiotic restriction alone is insufficient and that broader sepsis-prevention strategies are needed.
